# Dramatic Effect of A Ring Size of Alicyclic α-Dioximate Ligand Synthons on Kinetics of the Template Synthesis and of the Acidic Decomposition of the Methylboron-Capped Iron(II) Clathrochelates

**DOI:** 10.3390/molecules26134019

**Published:** 2021-06-30

**Authors:** Alexander L. Pomadchik, Alexander S. Belov, Ekaterina G. Lebed, Irina G. Belaya, Anna V. Vologzhanina, Yan Z. Voloshin

**Affiliations:** 1 Nesmeyanov Institute of the Organoelement Compounds of the Russian Academy of Sciences, 119991 Moscow, Russia; pom17@yandex.ru (A.L.P.); as_belov@mail.ru (A.S.B.); lebed@ineos.ac.ru (E.G.L.); mair2005@yandex.ru (I.G.B.); vologzhanina@mail.ru (A.V.V.); 2 Kurnakov Institute of General and Inorganic Chemistry of the Russian Academy of Sciences, 119991 Moscow, Russia

**Keywords:** macrocyclic compounds, cage complexes, clathrochelates, iron complexes, template synthesis, acidic decomposition, kinetics of complexation, thermodynamics of complexation, X-ray diffraction

## Abstract

Kinetics and thermodynamics of the template synthesis and of the acidic decomposition of the methylboron-capped iron(II) tris-1,2-dioximates—the clathrochelate derivatives of six (nioxime)- and eight (octoxime)-membered alicyclic ligand synthons—were compared. In the case of a macrobicyclic iron(II) tris-nioximate, the plausible pathway of its formation contains a rate-determining stage and includes a reversible formation of an almost trigonal-antiprismatic (TAP) protonated tris-complex, followed by its monodeprotonation and addition of CH_3_B(OH)_2_. Thus, the formed TAP intermediate undergoes a multistep rate-determining stage of double cyclization with the elimination of two water molecules accompanied by a structural rearrangement, thus giving an almost trigonal-prismatic (TP) iron(FII) semiclathrochelate. It easily undergoes a cross-linking with CH_3_B(OH)_2_, resulting in the elimination of H^+^ ion and in the formation of a macrobicyclic structure. In contrast, the analogous scheme for its macrobicyclic tris-octoximate analog was found to contain up to three initial stages affecting the overall synthesis reaction rate. The rates of acidic decomposition of the above clathrochelates were found to be also affected by the nature of their ribbed substituents. Therefore, the single crystal XRD experiments were performed in order to explain these results. The difference in the kinetic schemes of a formation of the boron-capped iron(II) tris-nioximates and tris-octoximates is explained by necessity of the substantial changes in a geometry of the latter ligand synthon, caused by its coordination to the iron(II) ion, due to both the higher distortion of the *FeN_6_*-coordination polyhedra, and the intramolecular sterical clashes in the molecules of the macrobicyclic iron(II) tris-octoximates.

## 1. Introduction

**Scheme 1:**
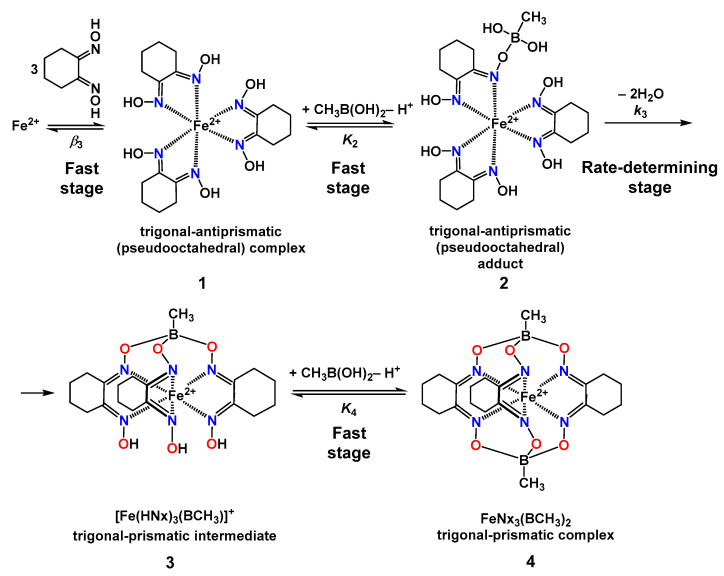
Most plausible pathway of a formation of the clathrochelate FeNx_3_(BCH_3_)_2_. Adapted from [6].

Reactions of the template synthesis of the boron-capped iron(II) cage complexes (clathrochelates [1,2]), the derivatives of six-, seven- and eight-membered alicyclic dioximes, are known [1,3,4,5,6] to be the unique objects thanks to experimental studies carried out on them. Indeed, the above clathrochelates quantitatively form in the diluted aqueous and/or aqueous–organic solutions in a wide range of the corresponding semiconversion reaction times, thus allowing their photometrical detection using the characteristic of this type of the quasiaromatic polyazomethine complexes intensive metal-to-ligand charge transfer (MLCT) Fed→Lπ* bands in the visible range with maxima at approximately 450 nm. Such experimental conditions seem to be more beneficial than those known from the literature [7,8,9,10,11,12,13,14] for their oximate and amine analogs. Recently, we performed [6] a detailed study of the kinetics and thermodynamics of both the template synthesis and the acidic decomposition of the macrobicyclic iron(II) tris-cyclohexanedion-1,2-dioximate (nioximate, Nx^2−^) FeNx_3_(BCH_3_)_2_ that is formed by a cross-linking with methylboronic acid. The proposed [6] pathway of its formation shown in Scheme 1 contains a multistep rate-determining stage and includes a reversible and fast formation of the protonated non-macrocyclic iron(II) tris-nioximate, possessing an almost TAP geometry with the TP–TAP distortion angle *φ* close to 60° (the *φ* value for an ideal TP is equal to 0° and that for an ideal TAP is equal to 60°). This first stage is so fast that the values of the corresponding direct *k*_1_ and reverse *k*_−1_ kinetic constants *(β*_3_ *= k*_1_*/k*_−1_) could not be obtained. It is followed by the monodeprotonation of **1** and the addition of the first molecule of CH_3_B(OH)_2_, giving the adduct **2**. The TAP intermediate obtained in this way undergoes a rate-determining stage of double cyclization with the elimination of two water molecules and an additional structural TAP→TP rearrangement. This gives a TP semiclathrochelate **3** that easily undergoes a cross-linking with the second molecule of CH_3_B(OH)_2_, resulting in the elimination of H^+^ ion and in the formation of the tris-nioximate iron(II) clathrochelate molecule **4** with two methylboron capping fragments [6]. In the present paper, we report the preparation of a new macrobicyclic iron(II) tris-α-dioximate; the derivative of an eight-membered alicyclic ligand synthon {cyclooctanedion-1,2 dioxime (octoxime), H_2_Ox} and the single crystal X-ray diffraction structures of this complex and also its above tris-cyclohexane-containing analog. We found a dramatic effect of the nature of an initial alicyclic α-dioxime on the kinetics and thermodynamics of both the template synthesis and the acidic decomposition of cage complexes under study.

(1) $$\begin{array}{l} \frac{dc}{dt}=k_{3}[Fe(H_{2}Nx{)}_{2}(HNx)\cdot CH_{3}B(OH{)}_{2}{}^{+}]=k_{3}K_{2}\beta_{3}[Fe^{2+}][H_{2}Nx{]}^{3}[CH_{3}B(OH{)}_{2}][H^{+}{]}^{-1}\\ k_{s}=k_{3}K_{2}\beta_{3} \end{array}$$

These changes in their parameters were explained using the obtained XRD data for them and those for their initial α-dioximate ligand synthons and clathrochelate analogs with known XRD structures.

## 2. Results and Discussion

### 2.1. Synthesis and Spectra

**Scheme 2:**
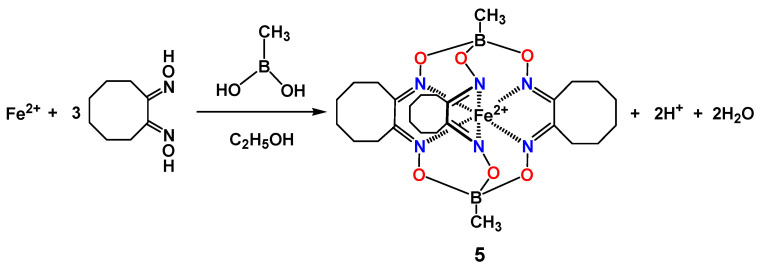
Preparation of the complex FeOx_3_(BCH_3_)_2_ (**5**).

Clathrochelate FeOx_3_(BCH_3_)_2_ (**5**) was obtained in a moderate yield of 65% by Scheme 2 under mild reaction conditions (i.e., at room temperature with ethanol as a solvent) using the template condensation of octoxime and methylboronic acid on the iron(II) ion as a matrix.

The prepared new iron(II) cage complex was characterized using elemental analysis, MALDI-TOF mass spectrometry, UV-Vis, ^1^H and ^13^C{^1^H} NMR spectra, as well as by the single crystal X-ray diffraction (see below). We also performed the analogous XRD experiment of its earlier-described [6] tris-nioximate analog FeNx_3_(BCH_3_)_2_ (**4**).

The diamagnetic character of these TP–TAP cage complexes, which have the low-spin *d^6^* electronic configuration of their encapsulated metal ion, is clearly seen from the NMR and XRD experiments (see below), as well as from the literature data [1,15,16,17,18] on ^57^Fe Mössbauer spectra for more than hundred iron(II) macrobicyclic tris-α-dioximates and tris-oximehydrazonates. Their three bonding *t*_2_*g → e*_1_ *+ a*_1_ orbitals are fully occupied [19,20,21,22,23,24,25] due to a very high ligand field strength, characteristic of a given type of the caging ligands. The experimentally observed increase in ligand field strength (as compared with the corresponding non-macrocyclic iron(II) tris-α-dioximates) can be due to the so-called “macrobicyclic effect” that is caused by a formation of their quasiaromatic macrobicyclic polyazomethine frameworks [1]. As a result, all these iron(II) cage complexes (such as the well-known pseudooctahedral, possessing an almost TAP geometry, iron(II) tris-phenanthrolinates and tris-α-bipyridinates [21,22,26,27,28]) are the low-spin diamagnetic compounds in their ground states.

The most intense peak in the positive range of MALDI-TOF mass spectrum of the clathrochelate FeOx_3_(BCH_3_)_2_ (**5**, see Supplementary Materials, Figure S8) belongs to its molecular ion. The number and positions of the signals in its solution ^1^H and ^13^C{^1^H} NMR spectra (see Supplementary Materials, Figures S9 and S10), as well as the ratios of their integral intensities in its ^1^H NMR spectrum (in particular, those of the signals of the apical methyl protons and of the *α-*, *β-* and *γ*-methylene protons of the ribbed eight-membered alicycles) confirmed the composition and *C*_3_-symmetry of the molecule FeOx_3_(BCH_3_)_2_ (**5**). It should also be noted that the signals of methylene protons in the solution ^1^H NMR spectra of the macrobicyclic complexes under discussion appeared in their spectra as broadened singlets (except that of α-methylene groups of new methylboron-capped iron(II) tris-octoximate, which appeared in its spectrum as a triplet) because their unsubstituted alicyclic fragments undergo a very fast exchange (in the NMR time scale at given experimental conditions) between their possible conformations. It should be noted that in the solution NMR spectra of more than hundred iron(II) clathrochelates—the derivatives of unsubstituted six-, seven- and eight-membered alicyclic α-dioximes, which have been prepared to date [1,2], we never observed a fine structure of the signals of protons of their α-, β- and γ-methylene groups, caused by the intramolecular ^1^H–^1^H interactions (except for those of α-methylene protons of the alkylboron-capped iron(II) eight-membered alicyclic α-dioximates). The above NMR spectra also suggest a diamagnetic character of new iron(II) complex FeOx_3_(BCH_3_)_2_ (**5**) (no paramagnetic shifts or paramagnetic broadenings of its NMR signals are observed). The number of lines in its ^13^C{^1^H} NMR spectrum also confirmed an equivalence of ribbed chelating α-dioximate fragments of its macrobicyclic molecule.

**Figure 1:**
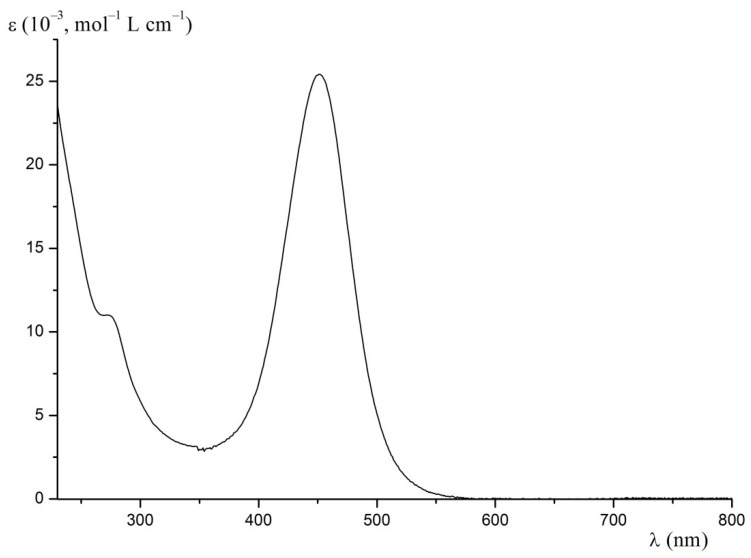
The solution UV-vis spectrum of the complex FeOx_3_(BCH_3_)_2_ (**5**) in dichloromethane at its concentration equal to 2.1 × 10^−4^ mol L^−1^.

UV-vis spectrum of the methylboron-capped tris-octoximate FeOx_3_(BCH_3_)_2_ (**5**) (Figure 1), measured from its solution in dichloromethane, contains in its visible range one intense (ε~2.6 × 10^4^ mol^−1^ L cm^−1^) asymmetric MLCT Fed→Lπ* band with maximum at approximately 450 nm.

### 2.2. Kinetics of the Template Synthesis of FeOx_3_(BCH_3_)_2_

Overall kinetic equation of the formation of this iron(II) cage complex (Scheme 2) can be described by Equation (2):

(2) $$\frac{dc}{dt}=k_{s}\cdot {\left[Fe^{2+}\right]}^{j}\cdot {\left[H_{2}Ox\right]}^{m}\cdot {\left[CH_{3}B{\left(OH\right)}_{2}\right]}^{n}\cdot {\left[H^{+}\right]}^{p}$$

At low concentrations of the iron(II) ions and a great excess of other reaction components (this suggests that the changes in their concentrations are negligible), this equation can be rewritten as:

(3) $$\frac{dc}{dt}=k_{eff}^{s}\cdot {\left[Fe^{2+}\right]}^{j}$$

where $k_{eff}^{s}=k_{s}\cdot {\left[H_{2}Ox\right]}^{m}\cdot {\left[CH_{3}B{\left(OH\right)}_{2}\right]}^{n}\cdot {\left[H^{+}\right]}^{p}$.

**Figure 2:**
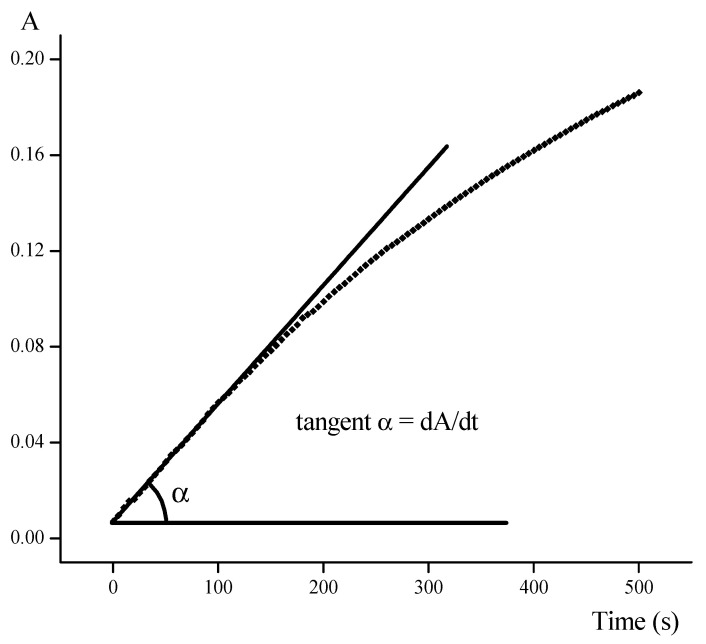
Example of a determination of the initial synthesis reaction rate *dA/dt* for the complex FeOx_3_(BCH_3_)_2_ (**5**) at [Fe^2+^] = 2 × 10^−5^ mol L^−1^, [H_2_Ox] = 1.5 × 10^−3^ mol L^−1^, [CH_3_B(OH)_2_] = 5 × 10^−3^ mol L^−1^ and pH = 3.75.

**Figure 3:**
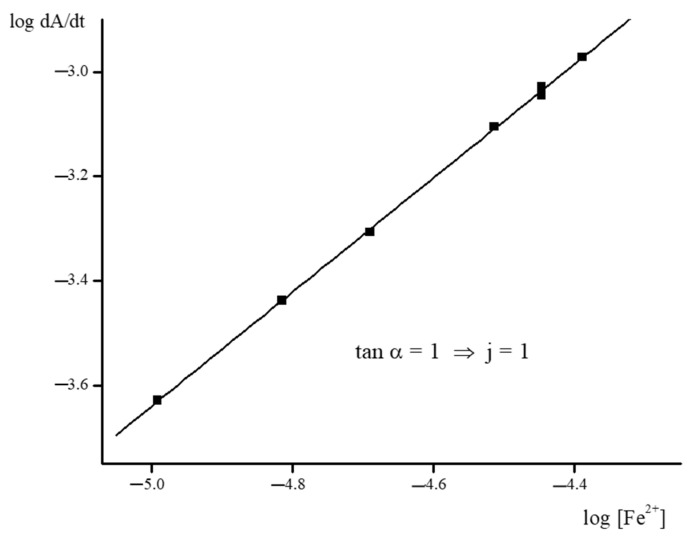
Determination of the synthesis reaction order for the complex FeOx_3_(BCH_3_)_2_ (**5**) with respect to the concentration of Fe^2+^ ions at [H_2_Ox] = 1.5 × 10^−3^ mol L^−1^, [CH_3_B(OH)_2_] = 5 × 10^−3^ mol L^−1^ and pH = 3.75.

So, it is possible to obtain an order with respect to the concentration of Fe^2+^ ions using a plot of the initial synthesis reaction rates *dc/dt* versus their concentration. Optical density *A* at a given wavelength after a given time interval is related to the concentration of the resulting clathrochelate FeOx_3_(BCH_3_)_2_ (**5**) by the classical equation: *A = ε l c* (where *ε* is the molar extinction coefficient of this complex, *c* is its molar concentration and *l* is the light path). Therefore, it is possible to use the *dA/dt* values as the characteristics of a synthesis reaction rate (Figure 2) and a set of the obtained experimental data can be described as the first-order kinetics (Equation (3)) with respect to the concentration of Fe^2+^ ions (see the plot of log(*dA/dt*) versus log[Fe^2+^] shown in Figure 3). 

Thus, the effective synthesis rate constant $k_{eff}^{s}$ at the low concentrations of Fe^2+^ ions was determined as a pseudo-first-order rate constant [29] using the following Equation (4):

(4) $$\ln\frac{A_{\max}}{A_{\max}-A}=k_{eff}^{s}\cdot t$$

where *A*_max_ is the maximal optical density that corresponds to a complete binding of Fe^2+^ ions caused by the formation of the complex FeOx_3_(BCH_3_)_2_ (**5**).

**Figure 4:**
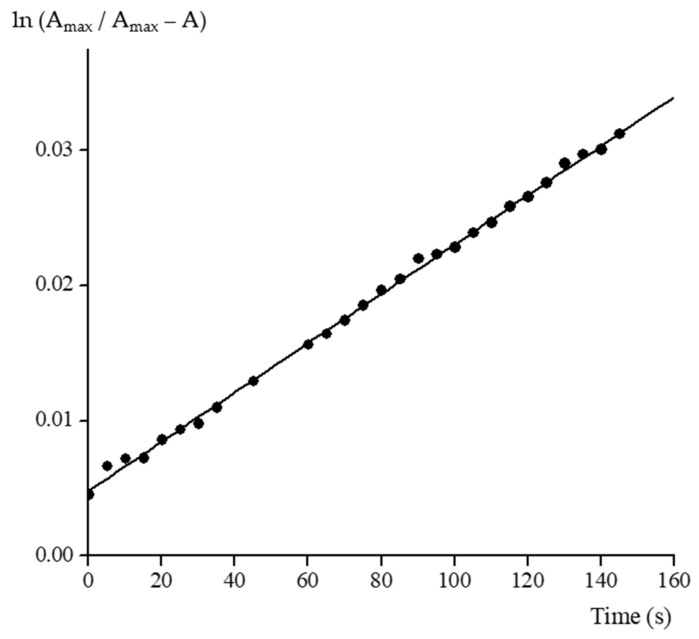
Example of an experimental determination of the synthesis reaction rate constant $k_{eff}^{s}$ for the complex FeOx_3_(BCH_3_)_2_ (**5**) at [Fe^2+^] = 2 × 10^−5^ mol L^−1^, [H_2_Ox] = 1.5 × 10^−3^ mol L^−1^, [CH_3_B(OH)_2_] = 5 × 10^−3^ mol L^−1^ and pH = 3.75.

Then the reaction orders with respect to the concentrations (or an activity in the case of the realizing H^+^ ions) of other components (H_2_Ox, CH_3_B(OH)_2_ and H^+^) were studied using the $k_{eff}^{s}$ values obtained as a slope of the corresponding linear regression, as shown in Figure 4.

**Figure 5:**
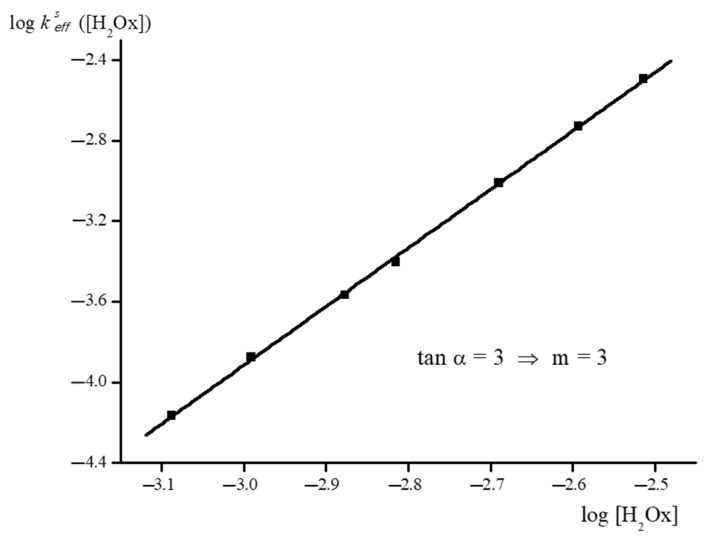
Determination of an order of the synthesis reaction rate constant $k_{eff}^{s}$ for the complex FeOx_3_(BCH_3_)_2_ (**5**) with respect to the concentration of octoxime at [Fe^2+^] = 1.5 × 10^−5^ mol L^−1^, [CH_3_B(OH)_2_] = 5 × 10^−3^ mol L^−1^ and pH = 3.3.

**Figure 6:**
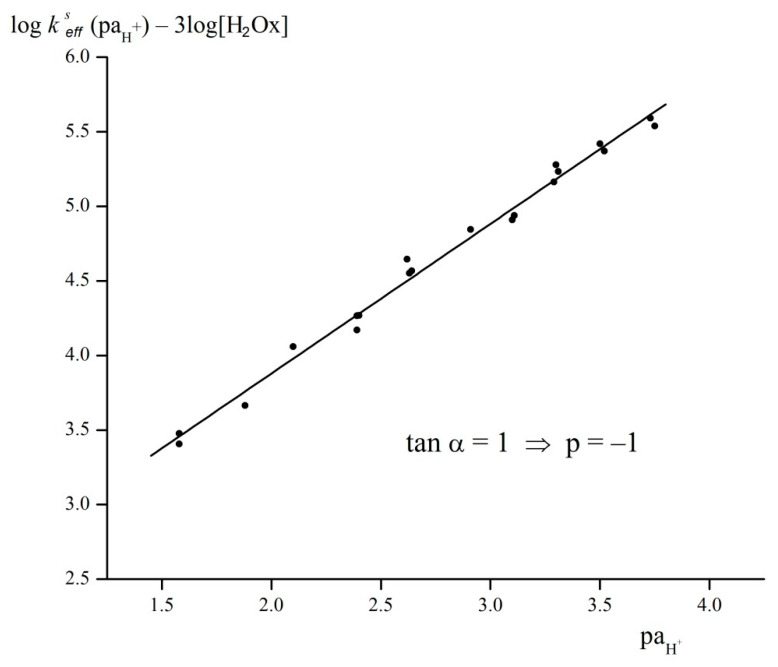
Plot of log$k_{eff}^{s}$($pa_{H^{+}}$) − 3 log[H_2_Ox] versus $pa_{H^{+}}$ for the complex FeOx_3_(BCH_3_)_2_ (**5**) at [Fe^2+^] = 2.5 × 10^−5^ mol L^−1^ and [CH_3_B(OH)_2_] = 1 × 10^−2^ mol L^−1^.

**Figure 7:**
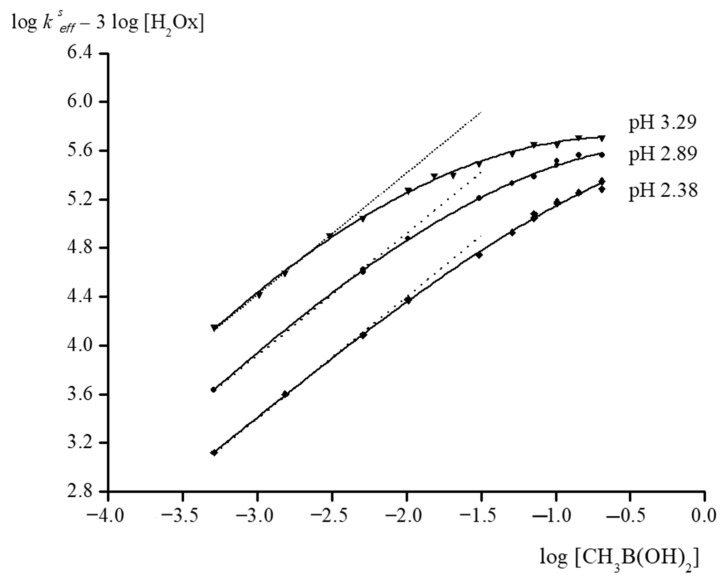
Plots of log$k_{eff}^{s}$ − 3 log[H_2_Ox] versus log[CH_3_B(OH)_2_] at the different pH values and various concentrations of Fe^2+^ ions and of octoxime.

To obtain a reaction order with respect to the concentration of a given component, its value has been changed in a relatively wide range, whereas the concentrations of other components persist. In the case of H^+^ ions, we suggested that in the diluted solutions, which were used for the kinetic experiments on the formation of a cage complex under study, the concentration of H^+^ ions could be assumed equal to their activity $a_{H^{+}}$. The latter value was measured using an appropriate pH-meter equipped with a given working pH-electrode (see the Experimental part). We suggested to obtain a reaction order with respect to the concentration of each of these components as the tangent of a linear regression plotted in the coordinates *log* $k_{eff}^{s}$ versus *log c_comp_*. Indeed, as it can be seen from Figure 5, such an order with respect to the concentration of octoxime is equal to 3. It was also found that at $pa_{H^{+}}$ values higher than approximately 3.8, the optical densities *A* of the solutions under study substantially increase, even in the absence of methylboronic acid. This effect is probably caused by the formation of the non-macrocyclic iron(II) mono- and bis-α-dioximates; the derivatives of a monodeprotonated form of octoxime (i.e., the HOx^−^ monoanion). Below this $pa_{H^{+}}$ value, the synthesis reaction order with respect to the activity of H^+^ ions is equal to –1 (Figure 6). On the other hand, in general, the corresponding plots versus the concentration of methylboronic acid (Figure 7) are not the linear regressions, but such a plot can adopt its linear form at the selected experimental conditions (first of all, at the appropriate concentrations of other components). 

A similar result has been previously observed [3] in the case of the hydroxyboron-capped macrobicyclic iron(II) tris-octoximate FeOx_3_(BOH)_2_. Indeed, the rates of two first stages of its formation are reported in this work to be comparable with that of a structural TAP→TP rearrangement, which is the rate-determining stage for other iron(II) clathrochelates of this type (Scheme 1). So, the Equations (5)–(7) describing three consecutive processes, the initial two of which are reversible, have been evaluated [3]. The rates of these intermediate stages have been assumed to be equal (i.e., the concentrations of the corresponding complex intermediates in the proposed [3] scheme of the formation of clathrochelate FeOx_3_(BOH)_2_ persist):

(5) $$dc/dt=k_{1}[Fe^{2+}]{\left[H_{2}Ox\right]}^{3}-k_{-1}[Fe{\left(H_{2}Ox\right)}_{3}^{2+}]$$

(6) $$\begin{array}{ll} dc/dt & =k_{2}[{Fe(H}_{2}{Ox)}_{3}^{2+}{][H}_{3}{BO}_{3}]-\\ & -k_{-2}[{Fe(H}_{2}{Ox)}_{2}(HOx)\cdot {B(OH)}_{3}^{+}{][H}^{+}] \end{array}$$

(7) $$dc/dt=k_{3}[{Fe(H}_{2}{Ox)}_{2}(HOx)\cdot {B(OH)}_{3}^{+}]$$

Solving these equations relative to the synthesis reaction rate, the following Equation (8), which is in good agreement with the obtained [3] experimental data, has been evaluated in that work:

(8) $$dc/dt=\frac{k_{3}k_{1}k_{2}[{Fe}^{2+}{][H}_{2}{Ox]}^{3}{[H}_{3}{BO}_{3}]}{k_{-1}k_{3}+k_{3}k_{2}[H_{3}{BO}_{3}]+k_{-1}k_{-2}[H^{+}]}$$

So, the analogous Equations (9)–(12) we used for evaluation of the obtained experimental results on a template assembling of the molecule FeOx_3_(BCH_3_)_2_ (**5**).

(9) $$dc/dt=k_{1}[{Fe}^{2}{}^{+}{][H}_{2}Ox{]}^{3}-k_{-1}[{Fe(H}_{2}{Ox)}_{3}^{2+}]$$

(10) $$\begin{array}{ll} dc/dt & =k_{2}[{Fe(H}_{2}{Ox)}_{3}^{2+}{][CH}_{3}{B(OH)}_{2}]-\\ & -k_{-2}[{Fe(H}_{2}{Ox)}_{2}(HOx)\cdot {CH}_{3}{B(OH)}_{2}^{+}{][H}^{+}] \end{array}$$

(11) $$dc/dt=k_{3}[{Fe(H}_{2}{Ox)}_{2}(HOx)\cdot {CH}_{3}{B(OH)}_{2}^{+}]$$

(12) $$dc/dt=\frac{k_{3}k_{1}k_{2}[{Fe}^{2+}{][H}_{2}{Ox]}^{3}{[CH}_{3}{B(OH)}_{2}]}{k_{-1}k_{3}+k_{3}k_{2}[{CH}_{3}{B(OH)}_{2}]+k_{-1}k_{-2}[H^{+}]}$$

The calculated ratios of the synthesis reaction rate constants for the complex FeOx_3_(BCH_3_)_2_ (**5**) thus allowed us to obtain the values of some constants in Equation (12) (in particular, those of the direct complexation Fe^2+^ + 3H_2_Ox → [Fe(H_2_Ox)_3_]^2+^ (*k*_1_) and a reverse decomplexation [Fe(H_2_Ox)_3_]^2+^ → Fe^2+^ + 3H_2_Ox (*k_−_*_1_) processes). For this purpose, we also used the initially experimentally obtained [3] value of the stability constant *β*_3_ for a given protonated non-macrocyclic iron(II) tris-octoximate **6**. To obtain values of those of the ratios, which are compiled in Table 1, Equation (12) was transformed into the following form:

(13) $$\frac{{[H_{2}Ox]}^{3}}{k_{eff}^{s}}=\frac{k_{-1}}{k_{1}k_{2}}(1+\frac{k_{-2}}{k_{3}}[H^{+}])\frac{1}{\left[{CH}_{3}{B(OH)}_{2}\right]}+\frac{1}{k_{1}}$$

Then, on the first stage of evaluation of Equation (13), we plotted the linear regressions of [H_2_Ox]^3^/$k_{eff}^{s}$ versus 1/[CH_3_B(OH)_2_] at various pH values (see Supplementary Materials, Figure S1), thus obtaining the values of 1/*k*_1_ as the corresponding Y-intercepts. The slopes of these linear regressions were used on the next stage of the experimental data treatment for obtaining of the *k_−_*_1_*/k*_2_ and *k_−_*_2_*/k*_3_ ratios from the corresponding linear regression versus the activity of H^+^ ions shown in Figure S3 (see Supplementary Materials) as its slope and its Y-intercept, respectively. It should be noted that values of *k*_1_ obtained in this way depend on the activity of H^+^ ions (see Supplementary Materials, Figure S2). This result can be explained by an increase in both an ionic strength (up to 9 × 10^−3^ mol L^−1^ at pH 2.38) and a dielectric constant. These two factors are known from the literature to substantially change the thermodynamic activities of polycharged inorganic and complex ions (in particular, those of the doubly charged metal cations). Therefore, they could also have an impact on the corresponding kinetic constants. Trying to take account of these effects in our further evaluation of the experimental data, the slopes of linear regressions versus 1/[CH_3_B(OH)_2_] (see Supplementary Materials, Figure S1), which were plotted on the above first step, we corrected on the second step of our calculations by dividing them by the values of the corresponding Y-intercepts. The plotted linear regression shown in Figure S3 (see Supplementary Materials) is well fitted to the obtained experimental data, which suggests that the amounts of both the above parameters (i.e., an ionic strength and a dielectric constant) in the solutions under study were precisely taken into account.

The *k*_−1_*k*_−2_[H^+^] value is much higher than that of *k*_−1_*k*_3_ + *k*_3_*k*_2_[CH_3_B(OH)_2_] in a wide range of the component’s concentrations and molar ratios. Therefore, Equation (12) assumes the regular for a given type of the boron-capped alicyclic iron(II) tris-α-dioximates form of Equation (14):

(14) $$dc/dt=k_{eff}^{s}[{Fe}^{2+}{][H}_{2}{D]}^{3}{\left[RB(OH\right)}_{2}{][H}^{+}{]}^{-1}$$

In particular, this equation is reported [5] to describe the synthesis reaction rate constants for the organoboron-capped tris-octoximate complexes FeOx_3_(BC_6_H_5_)_2_ and FeOx_3_(B*n*-C_4_H_9_)_2_. The observed changes of the kinetic schemes and thermodynamic parameters in a passing from the hydroxylboron-capped iron(II) tris-octoximate to its *n*-butyl- and phenylboron-cross-linked macrobicyclic analogs have been explained [5] by a substantial decrease in the rate constant *k*_−2_ of a reverse detachment reaction of a molecule of the corresponding organoboron Lewis acid. This results in an increase in the corresponding equilibrium constant *K*_2_ and, therefore, in the synthesis reaction rate constant *k_s_* as well (see Scheme 1 and Equation (1)). As a result, if the plausible scheme of a formation of the hydroxyboron-cross-linked complex FeOx_3_(BOH)_2_ contains at least two or three stages possessing the similar rates and thus affecting the overall synthesis reaction rate. In contrast, those for its above organoboron-capped analogs contain a rate-determining stage in the used ranges of their component’s concentrations. 

**Scheme 3:**
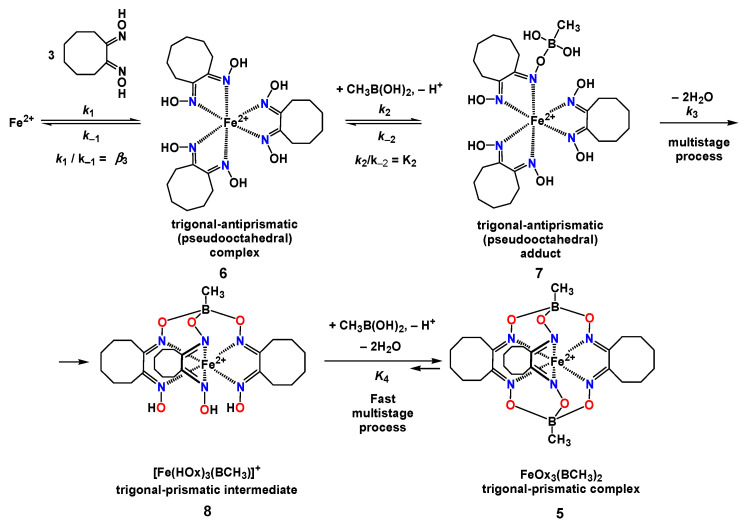
Plausible pathway of a formation of the clathrochelate FeOx_3_(BCH_3_)_2_ (**5**).

Similar deductions can be pointed out for explanation of the unexpected plots describing the experimental data on the synthesis reaction of the methylboron-capped macrobicyclic tris-octoximate FeOx_3_(BCH_3_)_2_ (**5**). Therefore, the plausible pathway of its formation shown in Scheme 3 can be evaluated. It includes a reversible formation of the protonated non-macrocyclic tris-octoximate dication [Fe(H_2_Ox)_3_]^2+^ (**6**), followed by a monodeprotonation of this TAP complex and by an addition of one molecule of methylboronic acid (or alternatively, vice versa, a deprotonation of CH_3_B(OH)_2_ and, then, an addition of its anionic form to **6** may take place), thus giving a TAP adduct **7**. Then, most probably, this adduct undergoes a double cyclization with the elimination of two water molecules to form the monocapped TP semiclathrochelate monocation **8**. A more detailed mechanism of this clearly multistage process may include, alternatively, a formation of the corresponding boronic hemiester on its second stage, and then, either a deprotonation of one oxime group of **7**—followed by its coordination to the Lewis-acidic boron atom possessing a trigonal arrangement in the molecule CH_3_B(OH)_2_, thus giving a tetrahedral O_3_BC group—or the molecule of this boronic acid forms the corresponding diester with **7**, which undergoes a following deprotonation. Both the above pathways shown in Scheme S1 (see Supplementary Materials) seem to be the probable chemical transformation. Indeed, the pK_a1_ value for methylboronic acid and those for the initial α-dioximes H_2_Nx and H_2_Ox, which have been measured in aqueous solutions, are known from the literature [30,31,32,33] to be almost equal and to fall in the ranges 10.4–10.7 [30,31], 10.6 [33] and 10.5–10.7 [32], respectively. So, at the maximal concentrations of the corresponding α-dioximes and CH_3_B(OH)_2_ equal to 3 × 10^−3^ and 3 × 10^−1^ mol L^−1^, respectively, and pH = 3.75, those of their monoanionic forms do not exceed 5 × 10^−10^ and 7 × 10^−8^ mol L^−1^, while the initial concentration of Fe^2+^ ions is close to 10^−5^ mol L^−1^. Moreover, dissociation of their molecules in mixed aqueous–organic media should be even lower. On the other hand, the non-macrocyclic metal oximate complexes typically possess substantially lower pK_a_ values than the initial oximes [34]. So, under the used experimental conditions, the proposed pathway of a given multistep chemical transformation shown in Scheme 3 is substantially more probable than that based on a formation of the monoanionic form of CH_3_B(OH)_2_ as the first stage (see Supplementary Materials, Scheme S1). Finally, the semiclathrochelate intermediate **8** formed can easily undergo a multistage cross-linking with the second molecule of methylboronic acid as a Lewis-acidic capping agent with elimination of H^+^ ion and two water molecules (see the discussion above), thus giving the dimethylboron-capped macrobicyclic molecule FeOx_3_(BCH_3_)_2_ (**5**) that possesses a distorted TP quasiaromatic cage framework (see below for its XRD structure). So, as follows from Scheme 3, which is in good agreement with the obtained experimental data, there are three main stages which determine the overall rate of the formation of this clathrochelate molecule.

### 2.3. X-ray Structures

Trying to explain the above differences in the kinetics of a formation of the clathrochelate analogs FeNx_3_(BCH_3_)_2_ (**4**) and FeOx_3_(BCH_3_)_2_ (**5**)—the derivatives of six- and eight-membered alicyclic α-dioximate ligand synthons—we performed the single crystal XRD study of their molecular structures. The latter were compared with known X-ray structures of the initial α-dioximes [35] and that of their *n*-butylboron-capped tris-octoximate analog FeOx_3_(B*n*-C_4_H_9_)_2_ [5].

**Figure 8:**
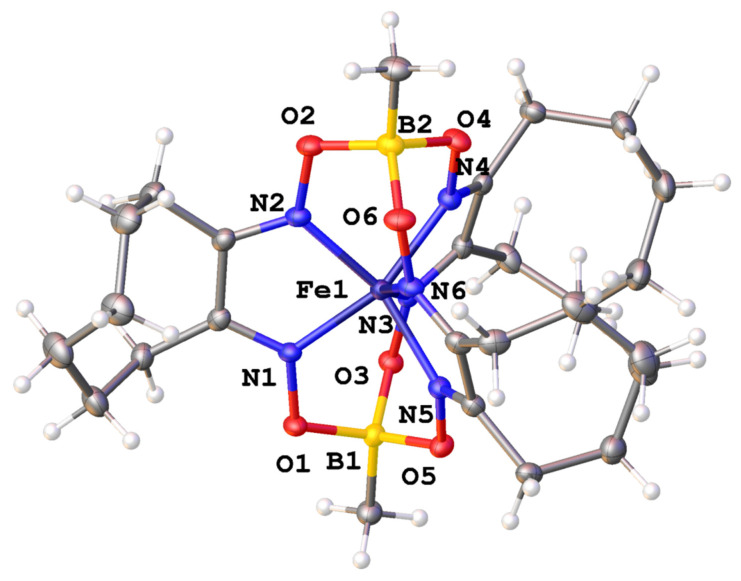
General view of the molecule FeOx_3_(BCH_3_)_2_ (**5**) in representation of its atoms with thermal ellipsoids (*p* = 50%).

**Figure 9:**
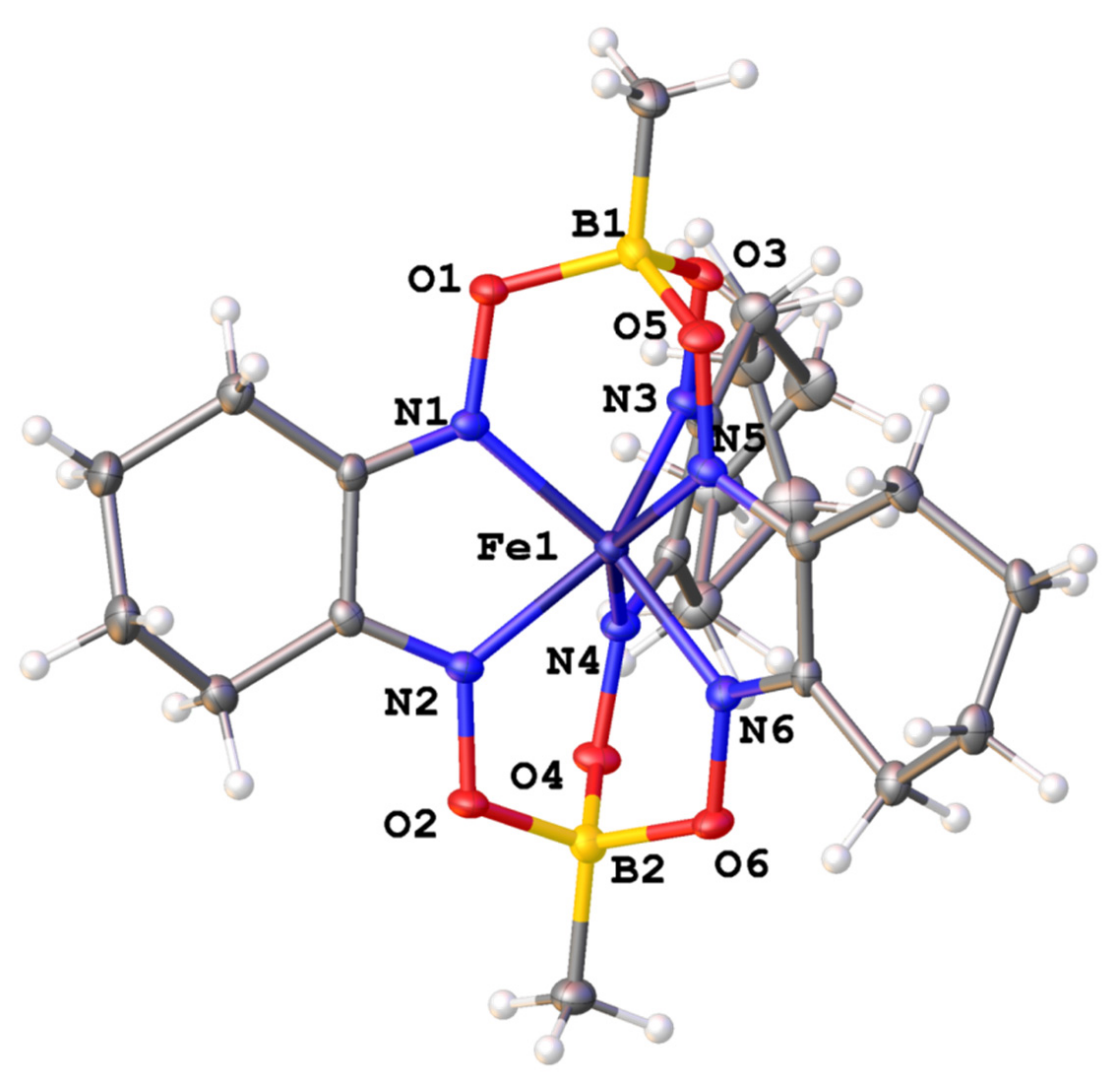
General view of the molecule FeNx_3_(BCH_3_)_2_ (**4**) in representation of its atoms with thermal ellipsoids (*p* = 50%).

General views of the molecules FeOx_3_(BCH_3_)_2_ (**5**) and FeNx_3_(BCH_3_)_2_ (**4**) are shown in Figure 8 and Figure 9, respectively; the main geometrical parameters of their clathrochelate frameworks and those for their *n*-butylboron- and adamantylboron-capped analogs are listed in Table 2 and Table 3. These tables also contain the direct XRD data for the molecules of the initial alicyclic α-dioximes. In the former macrobicyclic molecules, the encapsulated iron(II) ion is situated in the center of its *FeN_6_*-coordination polyhedron possessing the geometry intermediate between a TP and a TAP; the values of *φ* for these methylboron-capped tris-α-dioximate clathrochelates are equal to 26.0 and 18.9°, respectively. As can be seen from Table 2 and Table 3, the *φ* values for *n*-butylboron- and adamantylboron-capped iron(II) tris-octoximates [5,36] are higher than those for their tris-nioximate analogs, while other geometrical parameters of their macrobicyclic frameworks are very similar. In particular, the averaged Fe–N distances in all these molecules are close to 1.90 Å, thus they are characteristic of the macrobicyclic iron(II) tris-α-dioximates [1,2], and their oligo- and polymeric derivatives [37,38,39,40,41,42,43,44,45,46,47,48,49] as well, which are reported to be the diamagnetic compounds. Indeed, Fe–N distances in the high-spin iron(II) complexes, the derivatives of *N*-donor ligands, are typically higher than 2.0 Å [50,51].

**Figure 10:**
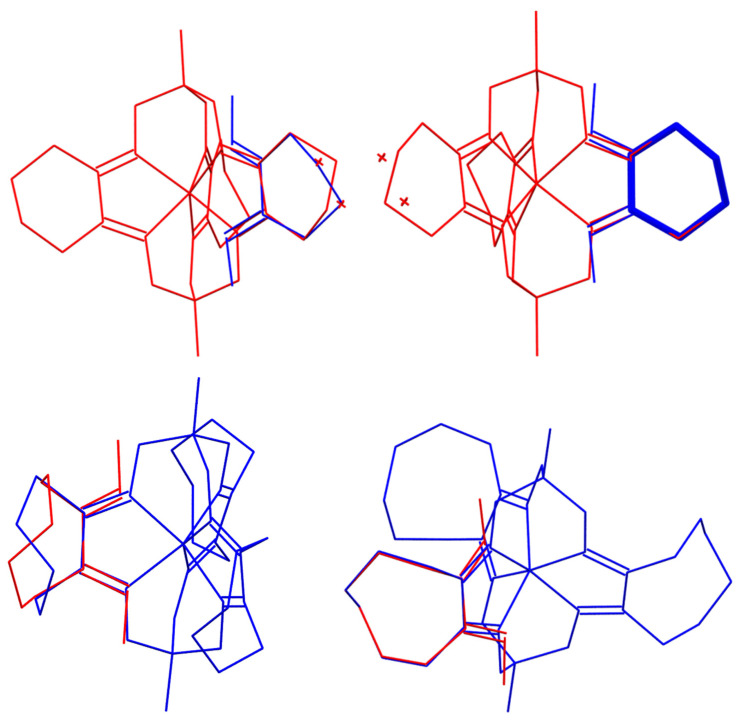
Comparison of a geometry of cyclohexane (on top) and cyclooctane (on bottom) alicyclic fragments in the corresponding boron-capped iron(II)-encapsulating clathrochelate molecules and the initial α-dioximes.

A relative rigidity of the π-conjugated diazomethine fragment N=C–C=N in the molecules of all alicyclic α-dioximes disfavors an ability of cyclohexane fragment of the initial nioxime molecule and those of its tris-α-dioximate clathrochelate derivatives to adopt its(their) *chair* or *boat* conformation(s). As a result, this(these) alicyclic fragment(s) adopt(s) a *twist* conformation in all cage molecules of this type with known X-ray structures, and in the molecule H_2_Nx as well. The average deviations of carbon atoms in its(their) six-membered alicyclic fragment(s) from the corresponding mean plain(s) do not exceed 0.10–0.13 Å (Figure 10, top). At the same time, its(their) structural lability causes a disordering of two α-carbon atoms of this six-membered alicycle, which are inherently bound to the above N=C–C=N fragment, into two crystallographically independent positions (i.e., the presence of two different *twist* conformations). Such a disordering is observed not only in the molecule FeNx_3_(BCH_3_)_2_ (**4**), but also in the X-rayed [53] co-crystals of nioxime with 4,4′-bipyridine and with 1,2-bis(4-pyridyl)ethane, as well as in the molecules of other boron-capped iron(II) tris-nioximates with known X-ray structures [1,5]. Despite the fact that the formation of a macrobicyclic framework does not affect the conformations of their alicyclic ribbed substituents, the presence of bulky aliphatic substituents at their capping boron atoms causes the substantial changes in the angles C=N–O and N=C–C=N in a rigid polyazomethine cage framework, and a shorting of the chelate C–C bonds as well (Table 2). At the same time, with other bond distances, valent and torsion angles in their macrobicyclic frameworks are characteristic of the corresponding structural elements, as follows from the “Mogul geometry check” treatments, which were implemented within the Mercury package [54].

**Figure 11:**
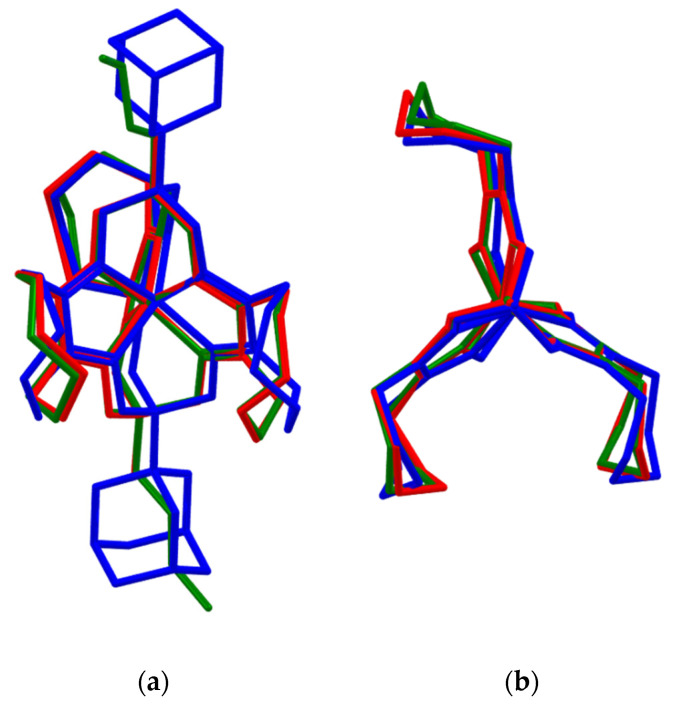
Superposition of the side (**a**) and top (**b**) views of the molecules FeOx_3_(BCH_3_)_2_ (**5**, shown in red), FeOx_3_(B*n*-C_4_H_9_)_2_ (shown in green) and FeOx_3_(BAd)_2_ (shown in blue) used for their comparison. Hydrogen atoms are omitted for clarity. Their main superimposed atoms are Fe1, B1, B2 and six nitrogen atoms as well.

It seems to be quite unexpected that, despite a big number of the rotational degrees of freedom in the case of an eight-membered alicyclic moiety, it adopts a *boat-chair* conformation (see Figure 10, bottom). The latter is fully ordered both in the initial molecule of octoxime [35], and in those of its clathrochelate derivatives with known X-ray structures (this work, [5,36]). Moreover, all these cage molecules, listed in Table 3, possess the very similar whole conformations: two of their three eight-membered alicyclic ribbed substituents are positioned close to each other (Figure 11). The changes in the mutual positions of oxygen, nitrogen and carbon atoms in the chains O–N=C–C=N–O of the molecule FeOx_3_(BCH_3_)_2_ (**5**), caused by their coordination to the encapsulated iron(II) ion, are similar to those for its tris-nioximate analogs. On the other hand, the average changes in the positions of carbon atoms of two cyclooctane rings of this molecule with respect to the molecule of initial α-dioxime are close to 0.05Å only, the third alicyclic fragment of this type underwent a complete inversion of its conformation, as compared with H_2_Ox. This effect can be caused by the formation of intramolecular interactions between two bulky neighboring eight-membered rings; the stabilization of an overall conformation of the molecule FeOx_3_(BCH_3_)_2_ (**5**) requires an adoption of the above fully inverted conformation for one of its three cyclooctane ribbed substituents (Figure 11). 

Thus, the observed differences in the kinetics and the schemes of a formation of the hydroxyboran- and methylboron-capped iron(II) tris-nioximates,—the derivatives of six-membered alicyclic α-dioxime—on the one hand, and those of their tris-octoximate analogs—the derivatives of eight-membered alicyclic ligand synthons—on the other hand, can be explained using the above direct XRD data. In the latter cases, the substantial structural changes of the geometry of their chelating ligand synthons, caused by their coordination to the encapsulated iron(II) ion are necessary. They can also result from a higher distortion of its TAP–TP *FeN_6_*-coordination polyhedron (and, therefore, that of a quasiaromatic cage framework), as well as from the intramolecular non-covalent interactions and sterical clashes of their bulky ribbed substituents in the molecules of the boron-capped macrobicyclic tris-octoximates. All these factors cause the substantial changes in the ratios between the rates of the multistep initial stages of these multistage template reactions, thus affecting their kinetic and thermodynamic parameters and their overall schemes as well (including the number of main stages which affect the overall synthesis reaction rate).

### 2.4. Acidic Decomposition of FeOx_3_(BCH_3_)_2_ in Its Diluted Solutions

The acidic decomposition rate constants $k_{eff}^{d}$ for the initially prepared complex FeOx_3_(BCH_3_)_2_ (**5**) were also obtained as the pseudo-first–order rate constants (Equation (15)), but with respect to the concentration of this clathrochelate:

(15) $$lnA=k_{eff}^{d}t$$

**Figure 12:**
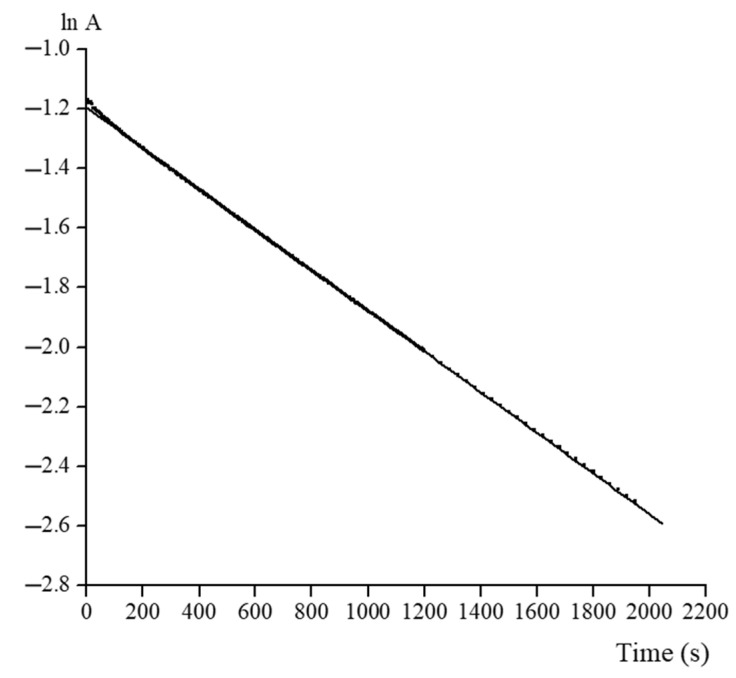
Example of a determination of the decomposition reaction rate constant $k_{eff}^{d}$ for the clathrochelate FeOx_3_(BCH_3_)_2_ (**5**) at its concentration 4.6 × 10^−5^ mol L^−1^ in 2.6 M solution of sulfuric acid ($a_{H^{+}}$ = 0.019 mol L^−1^).

An example of the determination of this constant is shown in Figure 12. The values of the decomposition reaction rate constants $k_{eff}^{d}$ were obtained as the tangents of linear regressions plotted in the coordinates lnA versus time.

**Scheme 4:**
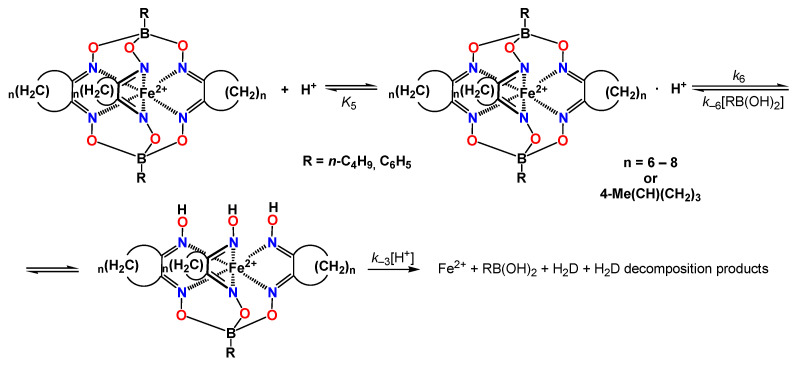
Most plausible pathway of the acidic decomposition of the *n-*butyl- and phenylboron-capped iron(II) alicyclic tris-α-dioximates. Adapted from [1].

Decomposition of the *n*-butyl- and phenylboron-capped iron(II) alicyclic tris-dioximates clathrochelates is known from literature [1,4,5,36] requiring their protonation (Scheme 4). The corresponding apical substituents cannot be protonated under these experimental conditions and H^+^ ions add to the ester oxygen atoms of the B–O–N chains of their capping fragment exclusively in the strongly acidic media. The following stepwise cleavage of the boron-containing capping groups and the protonation of the intermediate products lead to the irreversible decomposition of a given clathrochelate molecule. The addition of great amount of the corresponding boronic acid substantially increases the rate of reverse cross-linking process on the second stage of this decapping reaction, thus causing a decrease in its rate.

When the rates of the individual stages of this decomposition are deduced to be equal (i.e., the concentrations of its intermediates are constant), Scheme 4 can be described using the following equations:

(16) $$-d[complex]/dt=k_{-3}[Fe(HD{)}_{3}{\left(BR\right)}^{+}]\;a_{H^{+}}$$

(17) $$-d[complex]/dt=k_{6}[{FeD}_{3}{\left(BR\right)}_{2}\cdot H^{+}]-k_{-6}[{Fe(HD)}_{3}{(BR)}^{+}][RB(OH{)}_{2}]$$

(18) $$K_{5}=\frac{\left[{FeD}_{3}{\left(BR\right)}_{2}\cdot H^{+}\right]}{[{FeD}_{3}{\left(BR\right)}_{2}]\;a_{H^{+}}}$$

Since the changes in an optical density are caused by destruction of all of the colored complex forms shown in Scheme 4, their total concentration can be written as follows:

(19) $$\left[complex]=[Fe(HD{)}_{3}{\left(BR\right)}_{2}]+[Fe(HD{)}_{3}{\left(BR\right)}_{2}\cdot H^{+}]+[{Fe(HD)}_{3}{(BR)}^{+}\right]$$

So, the decomposition effective rate constant can be described as:

(20) $$k_{eff}^{d}=\frac{k_{-3}k_{6}K_{5}a_{H^{+}}^{2}}{k_{-3}a_{H^{+}}+k_{-6}[RB{\left(OH\right)}_{2}]+k_{-3}K_{5}a_{H^{+}}^{2}+k_{-6}K_{5}a_{H^{+}}[RB{\left(OH\right)}_{2}]+k_{6}K_{5}a_{H^{+}}}$$

This enables one to determine the values of the corresponding constants or those of their ratios. In the absence of the corresponding boronic acid and in harsh acidic media, the amounts of *k*_–6_[RB(OH)_2_] and of *K*_5_*k*_–6_$a_{H^{+}}$[RB(OH)_2_] become negligible, as compared with those of other components of this equation, and $k_{eff}^{d}$ can be expressed as:

(21) $$k_{eff}^{d}=\frac{k_{-3}k_{6}K_{5}a_{H^{+}}^{2}}{k_{-3}a_{H^{+}}+k_{6}K_{5}+k_{-3}K_{5}a_{H^{+}}}$$

**Figure 13:**
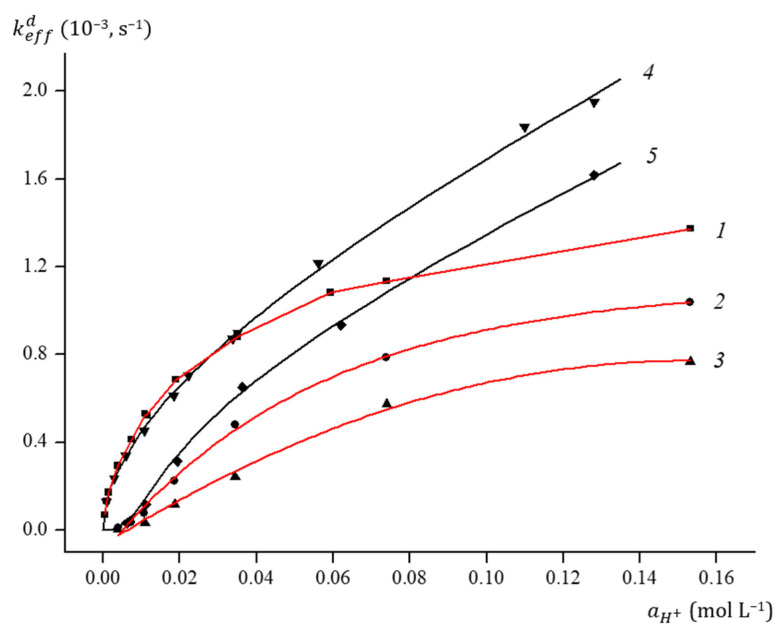
Plots of the $k_{eff}^{d}$ values for clathrochelates FeOx_3_(BCH_3_)_2_ (**5**, this work, shown in red, at the concentrations of methylboronic acid equal to 0 (*1*), 3.06 × 10^−2^ (*2*) and 6.38 × 10^−2^ (*3*) mol L^−1^) and FeNx_3_(BCH_3_)_2_ (**4**, shown in black, at the concentrations of methylboronic acid equal to 0 *(4)* and 1.53 × 10^−1^ *(5)* mol L^−1^) versus the activity of H^+^ ions. Adapted from ref. [6].

**Figure 14:**
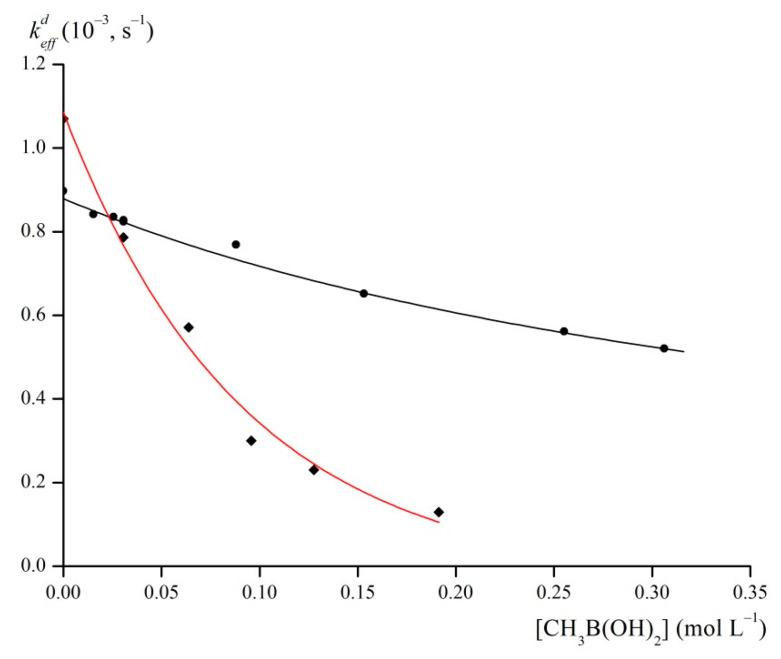
Plots of $k_{eff}^{d}$ of the acidic decomposition of clathrochelates FeOx_3_(BCH_3_)_2_ (this work, **5**, shown in red) and FeNx_3_(BCH_3_)_2_ (**4**, shown in black) versus the concentration of methylboronic acid at the activities of H^+^ ions equal to 7.39 × 10^−2^ and 3.62 × 10^−2^ mol L^−1^, respectively. Adapted from ref. [6].

Acidic decomposition of the cage iron(II) complexes under study both in the absence and in the presence of methylboronic acid (Figure 13 and Figure 14) could not be described by the above Equations (20) and (21). As can be seen from Figure 14, the values of $k_{eff}^{d}$ for the complexes FeNx_3_(BCH_3_)_2_ (**4**) and FeOx_3_(BCH_3_)_2_ (**5**) in the absence of methylboronic acid are almost the same up to the activity of H^+^ ions of approximately 0.035 mol L^−1^. On the other hand, its further increase resulted in a substantial difference between them (up to 30 ÷ 40% at the $a_{H^{+}}$ values in the range 0.13–0.15 mol L^−1^). Indeed, the corresponding $k_{eff}^{d}$ values for the tris-octoximate clathrochelate FeOx_3_(BCH_3_)_2_ (**5**) go up substantially slower than those for its tris-nioximate analog FeNx_3_(BCH_3_)_2_ (**4**). The addition of methylboronic acid caused a substantial decrease in the rates of their acidic decomposition (Figure 14), but this effect is more pronounced in the case of the complex FeOx_3_(BCH_3_)_2_ (**5**) than that for its homolog FeNx_3_(BCH_3_)_2_ (**4**). In particular, at the concentration of methylboronic acid equal to 3.06 × 10^−2^ mol L^−1^, the difference in the rates of the acidic decomposition of FeOx_3_(BCH_3_)_2_ (**5**) in the absence and in the presence of this capping agent is 0.3–0.4 10^−3^ s^−1^ (approximately 30–50%). The same effect for its tris-nioximate analog was observed at the concentration of methylboronic acid equal to 0.153 mol L^−1^. This suggests a substantial difference in the rates of the corresponding capping–decapping reactions. 

Moreover, we studied an effect of the addition of this Lewis-acidic capping agent on the decomposition of FeOx_3_(BCH_3_)_2_ (**5**). At the concentrations of CH_3_B(OH)_2_ higher than approximately 0.19 mol L^−1^ (at the activity of H^+^ ions equal to 7.39 × 10^−2^ mol L^−1^), the order of a given reaction with respect to the concentration of the initially obtained complex FeOx_3_(BCH_3_)_2_ (**5**) substantially increases, and this process already cannot be described using the known [29] pseudo-first-order kinetic equation. 

So, the following general Equation (22), describing all the obtained photometrical experimental data, was evaluated for the kinetics of the acidic decomposition of this cage complex versus the concentration of methylboronic acid and the activity of H^+^ ions as well:

(22) $$k_{eff}^{d}=\frac{a_{H^{+}}^{2}}{(E\cdot a_{H^{+}}+D\cdot a_{H^{+}}\cdot [CH_{3}B{\left(OH\right)}_{2}]+C\cdot a_{H^{+}}^{–0.5})\cdot [CH_{3}B{\left(OH\right)}_{2}]+B\cdot a_{H^{+}}^{2}+A\cdot a_{H^{+}}^{1.3}}$$

where *E* = 345, *D* = 12.3 × 10^3^, *C* = 3.33, *B* = 549 and *A* = 55.7.

For a more clear comparison, the earlier-evaluated [6] analogous equation for its homolog FeNx_3_(BCH_3_)_2_ (**4**) was transformed into the following form:

(23) $$k_{eff}^{d}=\frac{a_{H^{+}}^{2}}{E\;a_{H^{+}}+(D\cdot a_{H^{+}}\cdot +C\cdot a_{H^{+}}^{–1})\cdot [CH_{3}B{\left(OH\right)}_{2}]+B\cdot a_{H^{+}}^{2}+A\cdot a_{H^{+}}^{1.5}}$$

where *E* = 1.41, *D* = 70, *C* = 3 × 10^−3^, *B* = –160.4 and *A* = 237.8.

The presence in Equation (22), describing of the decomposition reaction of FeOx_3_(BCH_3_)_2_ (**5**), of the integer second order on the concentration of methylboronic acid allows us to suggest that the detachment of the second molecule of CH_3_B(OH)_2_ in harsh acidic media is also a reversible process. However, as with the case of its clathrochelate analog FeNx_3_(BCH_3_)_2_ (**4**), the presence of a fractional order with respect to the activity of H^+^ ions in this equation suggests the absence of its physical meaning. It is probable that at least two parallel pathways of its acidic decomposition, which are affected by addition of CH_3_B(OH)_2_, proceed under our experimental conditions.

## 3. Experimental

### 3.1. Synthesis

#### 3.1.1. Materials and Methods

The reagents used for the synthesis of a new clathrochelate complex under study, FeCl_2_·4H_2_O (Sigma-Aldrich^®^, St. Louis, MO, USA), methylboronic acid (Acros^®^, Tokyo, Japan), sorbents and organic solvents (Sigma–Aldrich^®^) were obtained commercially. Cyclooctanedion-1,2-dioxime (octoxime) was prepared by oxidation of cyclooctanone with SeO_2_ and the obtained alicyclic eight-membered α-diketone was treated with hydroxylamine [3]. The complex FeNx_3_(BCH_3_)_2_ (**4**) was obtained as described elsewhere [6]; its NMR and mass spectra are shown in (see Supplementary Materials, Figures S5–S7).

Analytical data (C, H, N contents) were obtained with a Carlo Erba model 1106 microanalyzer.

MALDI-TOF mass spectrum of the clathrochelate FeOx_3_(BCH_3_)_2_ (Figure S8) was recorded with and without the matrix using a MALDI-TOF-MS Bruker Autoflex II (Bruker Daltonics) mass spectrometer in reflecto-mol mode. The ionization was induced by UV-laser with wavelength 337 nm. The sample was applied to a nickel plate, and 2,5-dihydroxybenzoic acid was used as the matrix. The accuracy of measurements was 0.1%. 

^1^H and ^13^C{^1^H} NMR spectra of the complex FeOx_3_(BCH_3_)_2_ (**5**) shown in Figures S9 and S10 were recorded from its CD_2_Cl_2_ solution with a Bruker Avance 600 spectrometer. The measurements were carried out using the residual signals of this deuterated solvent.

UV-Vis spectrum of its solution in dichloromethane was recorded in the range 250–800 nm with a Varian Cary 100 spectrophotometer.

#### 3.1.2. Synthesis and Spectral Characteristics

FeOx_3_(BCH_3_)_2_ (**5**). Octoxime (0.22 g, 1.32 mmol) and FeCl_2_·4H_2_O (0.08 g, 0.40 mmol) were dissolved in ethanol (3 mL) and the reaction mixture was stirred for 10 min. Then methylboronic acid (0.05 g, 0.88 mmol) and ethanol (1 mL) were added and the reaction mixture was stirred for 2 h at r.t. The orange precipitate formed was filtered off, washed with ethanol (20 mL, in four portions) and extracted with dichloromethane (10 mL). The extract was filtered and chromatographically separated on silica gel (30-mm layer, eluent: dichloromethane). The first major red-orange elute was collected, filtered, the filtrate was evaporated to dryness, washed with hexane and dried in vacuo. Yield: 0.16 g (65 %). Found (%): C, 50.88; H, 7.02; N, 13.54. Calc. for C_26_H_42_N_6_B_2_FeO_6_ (%): C, 51.02; H, 6.92; N, 13.73. MS(MALDI-TOF): *m/z*: 612 [M]^+^^●^. ^1^H NMR (CD_2_Cl_2_, δ, ppm): –0.04 (br s, 6H, BCH_3_), 1.35 (br s, 12H, γ-CH_2_), 1.63 (br s, 12H, β-CH_2_), 2.92 (br t, JH1−H1= 6Hz, 12H, α-CH_2_). ^13^C{^1^H} NMR (CD_2_Cl_2_, δ, ppm): 25.83 (s, γ-CH_2_), 26.48 (s, β-CH_2_), 28.47 (s, α-CH_2_), 156.89 (s, C=N). 

#### 3.1.3. Single Crystal X-ray Diffraction Experiments

Single crystals of the complexes FeNx_3_(BCH_3_)_2_ (**4**) and FeOx_3_(BCH_3_)_2_·CH_2_Cl_2_ (**5**·CH_2_Cl_2_) were grown from their saturated solutions in chloroform:ethanol 1:10 and dichloromethane:heptane 1:3 mixtures, respectively. The intensities of reflections were measured with a Bruker Apex II CCD diffractometer using Mo–Kα radiation of a graphite monochromator (*λ* = 0.71073 Å). The structures were solved using the SHELXTL algorythm [55] and refined by full-matrix least squares against *F^2^*. Non-hydrogen atoms were refined in an anisotropic approximation; the positions of the H(C) atoms were calculated. All hydrogen atoms were included in a refinement by the riding model with *U_iso_*(H) = *nU_eq_*(X), where *n* = 1.5 for hydrogen atoms of methyl groups and 1.2 for other hydrogen atoms. All calculations were made using the SHELXL-2015 [56] and OLEX2 [57] program packages. The crystallographic parameters and the refinement details for the above X-rayed single crystals are listed in Table S1 (see Supplementary Materials).

CCDCs 2,036,146 and 2,036,147 contain the supplementary crystallographic data for this paper. These data can be obtained free of charge via http://www.ccdc.cam.ac.uk/structures, accessed on 18 May 2021.

### 3.2. Kinetic Studies

#### 3.2.1. Materials and Methods

The commercial FeSO_4_·7H_2_O (ACS Reagent, Sigma-Aldrich^®^), methylboronic acid (Acros^®^) and H_2_SO_4_ (Acros^®^) were used for the kinetic experiments; octoxime was obtained as described above. Bidistillated water and the additionally re-distilled ethanol and chloroform were used as the solvents for preparation of the reaction mixtures.

The initial reagents are almost optically silent in the visible range (see Supplementary Materials, Figure S4). So, kinetics of the template synthesis of FeOx_3_(BCH_3_)_2_ (**5**) and that of its acidic decomposition were studied using the photometric measurements at a maximum of the corresponding intense MLCT band (λ_max_ = 450 nm) with a Cary-50 UV-vis spectrophotometer equipped with a thermostated 1 × 1-cm quartz cell. Kinetic plots for both the synthesis and the acidic decomposition reactions of this macrobicyclic iron(II) tris-octoximate were evaluated using the “Kinetics” program package for the above spectrophotometer. The $a_{H^{+}}$ values for the diluted slightly acidic solutions were obtained using a Sartorius PP-20 pH-meter equipped with PY-P11 pH-electrode. The obtained kinetic data were evaluated using the Excel and Origin Pro program packages.

#### 3.2.2. Kinetic Experiments

Cage iron(II) complex FeOx_3_(BCH_3_)_2_ (**5**) was formed in the quantitative yield in the diluted water–ethanol–chloroform mixture at 25 °C. The diluted aqueous solutions of FeSO_4_·7H_2_O and sulfuric acid were mixed with those of methylboronic acid and octoxime in ethanol for studying of kinetics of its synthesis. Chloroform (10 vol.%) was also added to increase solubility of this complex in a given solvent mixture. In all cases, the water:ethanol:chloroform *v/v* ratio was 3:6:1.

Kinetics of the acidic decomposition of the initially obtained clathrochelate FeOx_3_(BCH_3_)_2_ (**5**) was studied using its solution in the same solvent mixture. A weighted amount of this complex, prepared as described above, was dissolved in a given volume of ethanol:chloroform 6:1 *v/v* mixture. Acidic decomposition experiments both in the absence and in the presence of methylboronic acid were performed using a bulk 13.3 ÷ 13.5M aqueous H_2_SO_4_ solution.

Decomposition of the complex FeOx_3_(BCH_3_)_2_ (**5**) proceeds within a reasonable time interval only in the strongly acidic media. Thus, the activities of H^+^ ions at high concentrations of H_2_SO_4_, as a source of these ions, in a given water–ethanol–chloroform mixture should be used in the corresponding kinetic and thermodynamic calculations. That is why we used the so-called “acidity function” H_0_ = –log $a_{H^{+}}$, the graphical presentation of which is plotted [6]; it has been earlier obtained [58] using an indicator method with *ortho*- and *para*-nitroanilines as UV-vis indicators in the same mixture at the same concentrations of H_2_SO_4_.

## 4. Conclusions

Thus, we unexpectedly found the dramatic effect of a ring size (six- versus eight-membered alicyclic substituents) of the chelating α-dioximate ligand synthons on the plausible kinetic scheme and thermodynamic parameters of the reactions of template assembly of their methylboron-capped iron(II)-encapsulating clathrochelate derivatives in the diluted aqueous–organic solutions. This factor also affects the rates of their decomposition in harsh acidic media. To explain the obtained results of the experimental solution photometric studies, we performed the single crystal XRD experiments for both these diamagnetic iron(II) clathrochelates and compared their results with known XRD structures of the initial alicyclic six- and eight-membered α-dioximes and those of their clathrochelate derivatives. This allowed us to deduce the structural reasons of the above substantial changes in the kinetics and thermodynamics of the synthesis and decomposition reactions of the macrobicyclic iron(II) compounds under study. The difference in the kinetic schemes of a formation of these alicyclic cage complexes was explained by necessity of the substantial changes in a geometry of the octoximate chelate fragments, caused by their coordination to the iron(II) ion, due to both the higher distortion of the *FeN_6_*-coordination polyhedra, and the intramolecular sterical clashes in the molecules of the macrobicyclic iron(II) tris-octoximates.

## Figures and Tables

**Table 1:** Kinetic and thermodynamic parameters of the template synthesis and the acidic decomposition reactions of the methylboron-capped iron(II) tris-octoximate FeOx_3_(BCH_3_)_2_ (**5**), and those for its tris-nioximate (**4**) [6] and hydroxy-, phenyl- and *n*-butylboron-capped tris-octoximate [1] analogs.

Complex	^b^ log*β*_3_	*k*_s_ s^−1^ L^3^ mol^−3^	*k*_1_ × 10^−5^ s^−1^ L^3^ mol^−3^	*k*_−1_ s^−1^	*k*_2_ s^−1^ L mol^−1^	*k*_−2_ */k*_3_	*k*_−3_ s^−1^ L^2^ mol^−2^	^c^ *K*_4_	*K*_5_	*k*_6_ × 10^5^ s^−1^ L^2^ mol^−2^	*k*_−6_ s^−1^ L^2^ mol^−2^
^a^ FeOx_3_(BCH_3_)_2_	^b^ 5.37		4.36 (pH2.38) 5.73 (pH3.18) 6.78 (pH3.75)	1.86 (pH2.38) 2.45 (pH3.18) 2.89 (pH3.75)	343 (pH2.38) 451 (pH3.18) 533 (pH3.75)	8960					
FeOx_3_(BOH)_2_	^b^ 5.37		0.445	0.19	22	424	0.12	129			
FeOx_3_(BC_6_H_5_)_2_		1.2 × 10^4^					0.09	292	240	5.0	3.5
FeOx_3_(B*n*-C_4_H_9_)_2_		2.1 × 10^4^					0.11	147	129	31.1	5.9
FeNx_3_(BCH_3_)_2_	^b^ 4.94	3.2 × 10^3^									

^a^ This work. ^b^ *β*_3_ is an earlier obtained [1] stability constant for the corresponding non-macrocyclic tris-dioximate [Fe(H_2_D)_3_]^2+^ measured [1] in aqueous solutions. ^c^ K_4_ = K_5_^−1^K_6_^−1.^

**Table 2:** Main geometrical parameters of the alkylboron-capped iron(II) tris-octoximate molecules and those for initial α-dioxime (octoxime).

Parameter	^a^ FeOx_3_(BCH_3_)_2_ (5)	FeOx_3_(B*n*-C_4_H_9_)_2_ [5]	^b^ FeOx_3_(BAd)_2_ [36]	H_2_Ox [35]
Fe–(Å)	1.910(2)–1.921(2)	1.868(11)–1.929(11)	1.895(2)–1.911(2)	-
	av. 1.916	av. 1.895	av. 1.899	
B–O (Å)	1.512(3)–1.526(3)	1.497(19)–1.510(20)	1.495(4)–1.515(4)	-
	av. 1.519	av. 1.505	av. 1.502	
N–O (Å)	1.387(2)–1.394(2)	1.363(13)–1.394(10)	1.366(3)–1.383(3)	1.409(2)–1.413(4)
	av. 1.390	av. 1.375	av. 1.375	av. 1.411
C=N (Å)	1.315(3)–1.323(2)	1.28(2)–1.34(2)	1.289(4)–1.313(4)	1.291(3)–1.293(7)
	av. 1.318	av. 1.314	av. 1.303	av. 1.292
chelate C–C (Å)	1.460(3)–1.463(3)	1.439(16)–1.459(17)	1.440(5)–1.454(5)	1.492(7)
	av. 1.461	av. 1.445	av. 1.447	
^c^ N…N (Å)	2.432(2)–2.436(3)	2.387(12)–2.412(14)	2.401(4)–2.408(4)	2.667(3)
	av. 2.434	av. 2.397	av. 2.405	
N=C–C=N (°)	av. 6.8	av. 5.7	av. 6.2	26.6(6)
φ (°)	26.0	25.1	25.2	
α (°)	78.8	78.4	78.6	
h (Å)	2.34	2.31	2.31	

^a^ This work. ^b^ “*Ad*” means an adamantyl residue. ^c^ “N…N” means the distance between two nitrogen atoms of the same α-dioximate ligand fragment.

**Table 3:** Main geometrical parameters of the alkylboron-capped iron(II) tris-nioximate molecules and those for the initial α-dioxime (nioxime).

Parameter	^a^ FeNx_3_(BCH_3_)_2_ (4)	FeNx_3_(B*n*-C_4_H_9_)_2_ [52]	^b^ FeNx_3_(BAd)_2_ [36]	H_2_Nx [35]
Fe–N (Å)	1.911(2)–1.918(2)	1.895(3)–1.923(3)	1.898(2)–1.906(2)	
	av. 1.914	av. 1.911	av. 1.902	
B–O (Å)	1.498(3)–1.513(3)	1.466(5)–1.532(5)	1.499(3)–1.506(3)	
	av. 1.503	av. 1.504	av. 1.503	
N–O (Å)	1.372(2)–1.379(2)	1.368(3)–1.386(3)	1.365(2)–1.374(2)	1.402(3)–1.410(3)
	av. 1.376	av. 1.376	av. 1.369	av. 1.406
C=N (Å)	1.302(3)–1.314(3)	1.295(5)–1.325(4)	1.304(3)–1.308(2)	1.276(3)–1.280(3)
	av. 1.308	av. 1.309	av. 1.306	av. 1.278
C–C (Å)	1.435(3)–1.446(3)	1.414(4)–1.441(4)	1.432(2)–1.439(3)	1.489(3)
	av. 1.440	av. 1.428	av. 1.436	
^c^ N…N (Å)	2.420(2)–2.424(3)	2.402(3)–2.427(3)	2.413(3)–2.425(2)	2.682(3)
	av. 2.421	av. 2.414	av. 2.417	
N=C–C=N (°)	av. 6.5	av. 7.6	av. 6.0	26.2(2)
φ (°)	18.9	21.1	22.9	
α (°)	78.4	78.4	79	
h (Å)	2.37	2.35	2.34	

^a^ This work. ^b^ “*Ad*” means an adamantyl residue. ^c^ “N…N” means the distance between two nitrogen atoms of the same α-dioximate ligand fragment.

## Data Availability

Data available in a publicly accessible repository.

## Supplementary Materials

The following materials are available online, Table S1. Crystallographic parameters and the experimental details for the X-rayed single crystals of the methylboron-capped alicyclic tris-α-dioximates FeNx_3_(BCH_3_)_2_ and FeOx_3_(BCH_3_)_2_·CH_2_Cl_2_. Scheme S1. Probable alternative pathways of a formation of the clathrochelate FeOx_3_(BCH_3_)_2_ (**5**). Figures S1–S4. Plots of linear regressions for determination of various kinetic constants. Figures S5–S10. The experimental NMR and mass spectra for the above cage complexes, and the discussion about their spin state.

## Sample Availability

Samples of the compounds are not available from the authors.
