# Supplementary material for: Dramatic Effect of A Ring Size of Alicyclic α-Dioximate Ligand Synthons on Kinetics of the Template Synthesis and of the Acidic Decomposition of the Methylboron-Capped Iron(II) Clathrochelates
Source: Molecules. 2021 Jun 30;26(13):4019. doi: 10.3390/molecules26134019 (PMC8271458; doi:10.3390/molecules26134019)
Supplement: Supplementary file 1 [file molecules-26-04019-s001.zip › molecules-1245643-supplementary.pdf]

Supplementary Materials

for

Dramatic effect of a ring size of alicyclic α-diox𝑖mate ligand's synthons on kinetics of the template synthesis and of the acidic decomposition of the methylboron-capped iron(II) clathrochelates

Alexander L. Pomadchik,[a] Alexander S. Belov,[a] Ekaterina G. Lebed,[a] Irina G. Belaya,[a] Anna V.Vologzhanina,[a] Yan Z. Voloshin [a,b,*]

[a] *Nesmeyanov Institute of the Organoelement Compounds of the Russian Academy of Sciences, 119991 Moscow, Russia*

[b] *Kurnakov Institute of General and Inorganic Chemistry of the Russian Academy of Sciences, 119991 Moscow, Russia*

**e-mails: voloshin@ineos.ac.ru, voloshin@igic.ras.ru*

Spin state of the encapsulated iron(II) ion in the cage complexes under study

The low-spin state of all the macrobicyclic iron(II) tris-α-dioximates can be clearly seen from the following experimental data:

1. from ^{57}Fe Mössbauer spectra for more than hundred complexes of this type (see, for example, Y.Z.Voloshin, N.A.Kostromina, R.Krämer, *Clathrochelates: Synthesis, Structure and Properties*, Elsevier, 2002 and references therein; Y.Z. Voloshin, E.V. Polshin, A.Y. Nazarenko, *Hyperfine Inter*. 2002, 141, 309–320 and references therein; Y.Z. Voloshin, O.A. Varzatskii, T.E. Kron, V.K. Belsky, V.E. Zavodnik, N.G Strizhakova, A.V. Palchik, *Inorg.Chem*., 2000, 39, 1907–1918; Y.Z. Voloshin, V.E. Zavodnik, O.A. Varzatskii, V.K. Belsky, A.V. Palchik, N.G. Strizhakova, I.I. Vorontsov, M.Y. Antipin, *Dalton Trans*. 2002, 1193–1202 etc).

2. from NMR data (the absence of any paramagnetic shifts or paramagnetic broadenings).

3. From the short values of their averaged Fe–N distances (approximately 1.90Å), as compared with those for the high-spin iron(II) complexes with nitrogen donor ligands (typically 2.1 – 2.2Å).

The experimentally observed increase in (as compared with the corresponding non-macrocyclic iron(II) tris-α-dioximates) and a very high ligand field strength characteristic of a given type of the iron(II) cage complexes [Y.Z. Voloshin, E.V. Polshin, A.Y. Nazarenko, *Hyperfine Inter*. 2002, 141, 309–320 and Y.Z.Voloshin, N.A.Kostromina, R.Krämer, *Clathrochelates: Synthesis, Structure and Properties*, Elsevier, 2002 and references therein] can be due to the so-called “macrobicyclic effect” that is caused by a formation of their quasiaromatic macrobicyclic polyazomethine ligands.

Table S1. Crystallographic parameters and the experimental details for the X-rayed single crystals of the methylboron-capped alicyclic tris-α-dioximates $FeNx_3(BCH_3)_2$ (**4**) and $FeOx_3(BCH_3)_2 \cdot CH_2Cl_2$ (**5**$\cdot CH_2Cl_2$)

**Parameter**	$FeNx_3(BCH_3)_2$ (**4**)	$FeOx_3(BCH_3)_2 \cdot CH_2Cl_2$ (**5**$\cdot CH_2Cl_2$)
Empirical formula	$C_{20}H_{30}B_2FeN_6O_6$	$C_{27}H_{44}B_2Cl_2FeN_6O_6$
Formula weight	537.97	697.05
Color, habit	Red, plate	Orange, prism
Crystal size (mm)	0.15 × 0.10 × 0.01	0.40 × 0.16 × 0.15
a (Å)	9.7952(7)	11.253(6)
b (Å)	9.8006(7)	11.512(7)
c (Å)	13.745(1)	13.357(13)
α (°)	71.168(2)	78.32(3)
β (°)	81.992(2)	86.10(3)
γ (°)	68.448(2)	74.29(2)
V (Å^3)	1161.23(15)	1631(2)
Z	2	2
Crystal system	triclinic	Triclinic
Space group	$P\bar{1}$	$P\bar{1}$
D_{cal} (g cm^{-3})	1.510	1.419
μ (mm^{-1})	0.700	0.676
Reflections collected	13567	20145
Independent reflections (R_{int})	5592 (0.052)	9136 (0.035)
Obs.refl./restraints/ parameters	4024 / 0 / 325	6834 / 0 / 399
$R,^a$ % [$F^2 > 2\sigma(F^2)$]	0.044	0.047
$R_w,^b$ % (F^2)	0.101	0.098
GOF	1.01	1.03
Largest diff. peak and hole (e Å^{-3})	0.47 and -0.31	0.77 and – 0.68
F(000)	552	732

$^aR = \Sigma||F_o| - |F_c||/\Sigma|F_o|$. $^bR_w = [\Sigma(w(F_o^2 - F_c^2)^2)/\Sigma(w(F_o^2))]^{1/2}$.

^{c}GOF = $[\Sigma w(F_o^2 - F_c^2)^2/(N_{obs} - N_{param})]^{1/2}$

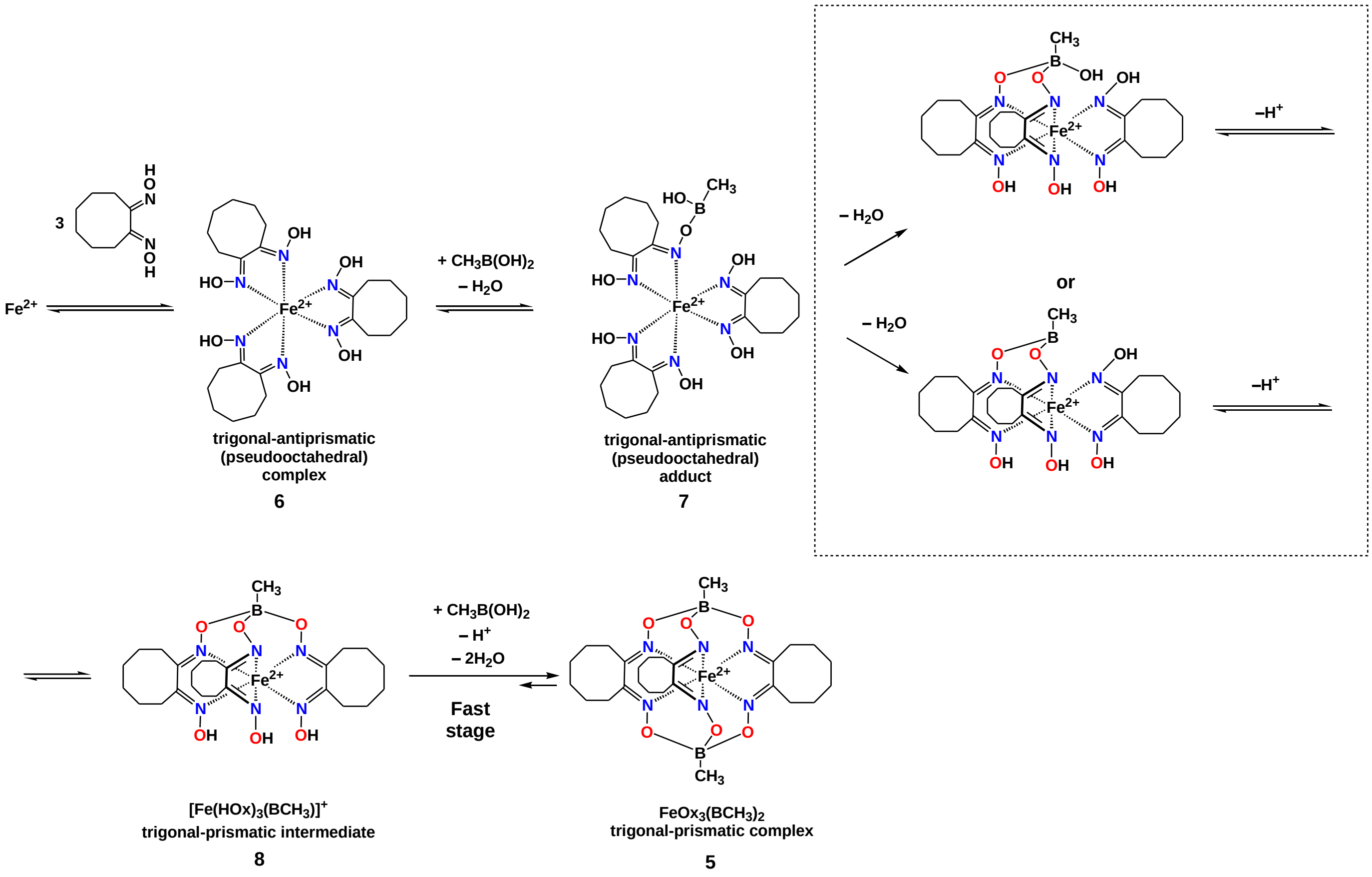

Scheme S1. Probable alternative pathways of a formation of the clathrochelate $FeOx_3(BCH_3)_2$ (**5**).

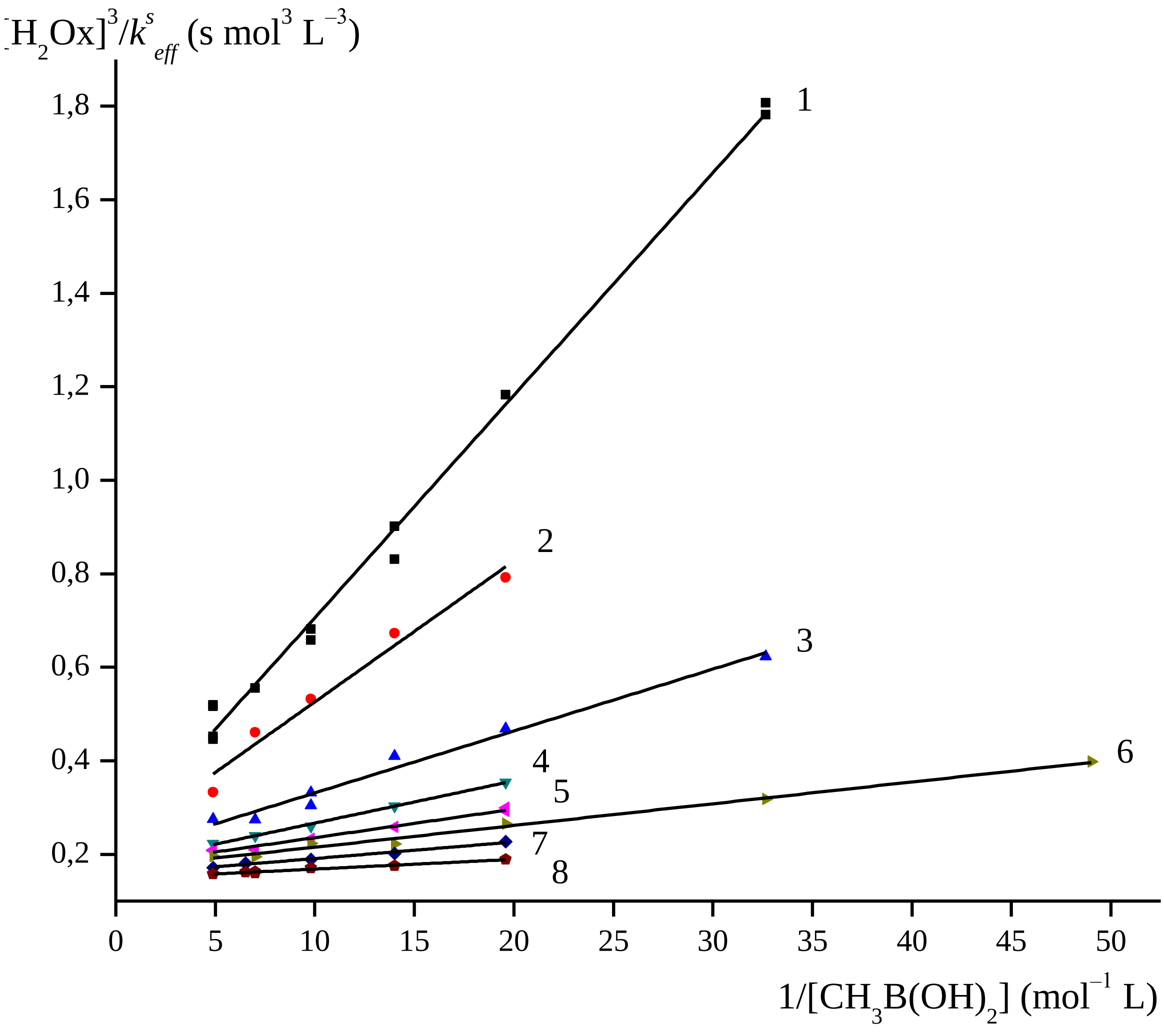

Figure S1. Determination of the synthesis reaction rate constants in Eq.13 at the pH values of 2.38 (*1*), 2.58 (*2*), 2.89 (*3*), 3.05 (*4*), 3.18 (*5*), 3.29 (*6*), 3.53 (*7*) and 3.75 (*8*).

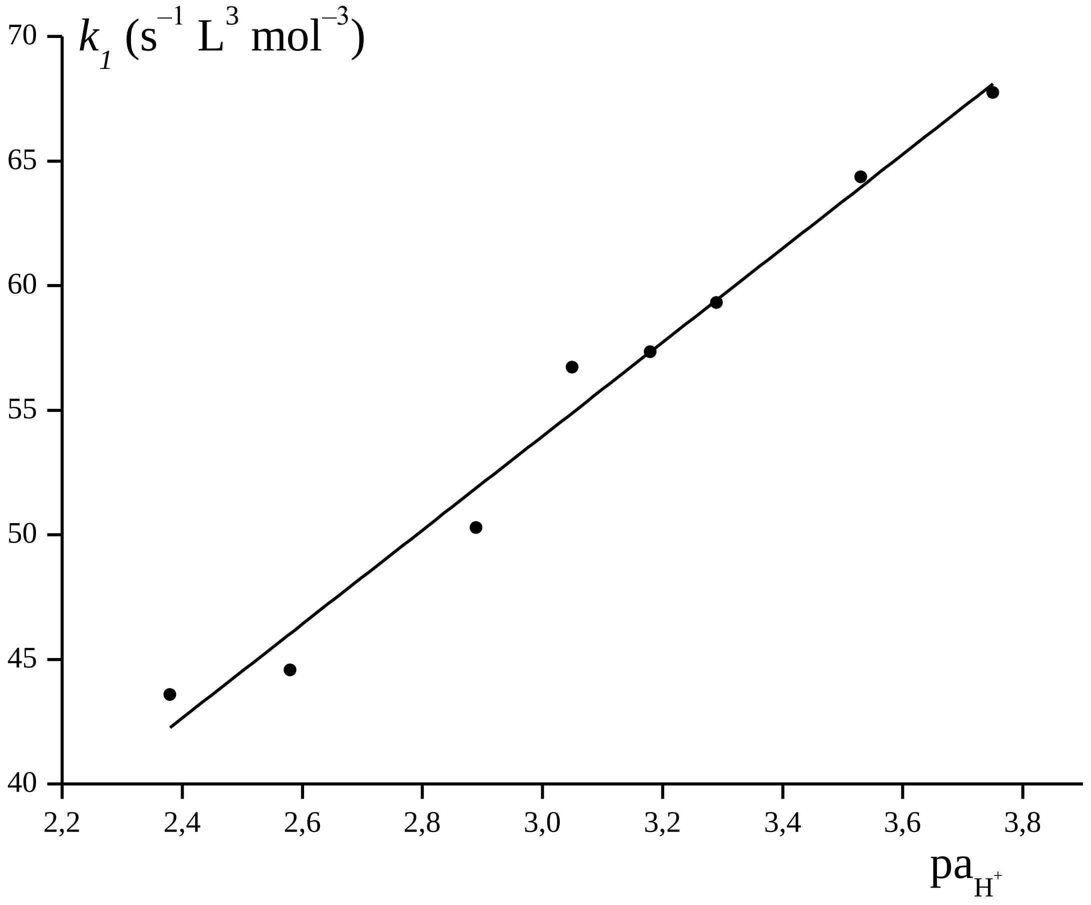

Figure S2. Plot of the k_1 value *versus* pa_{H^+}.

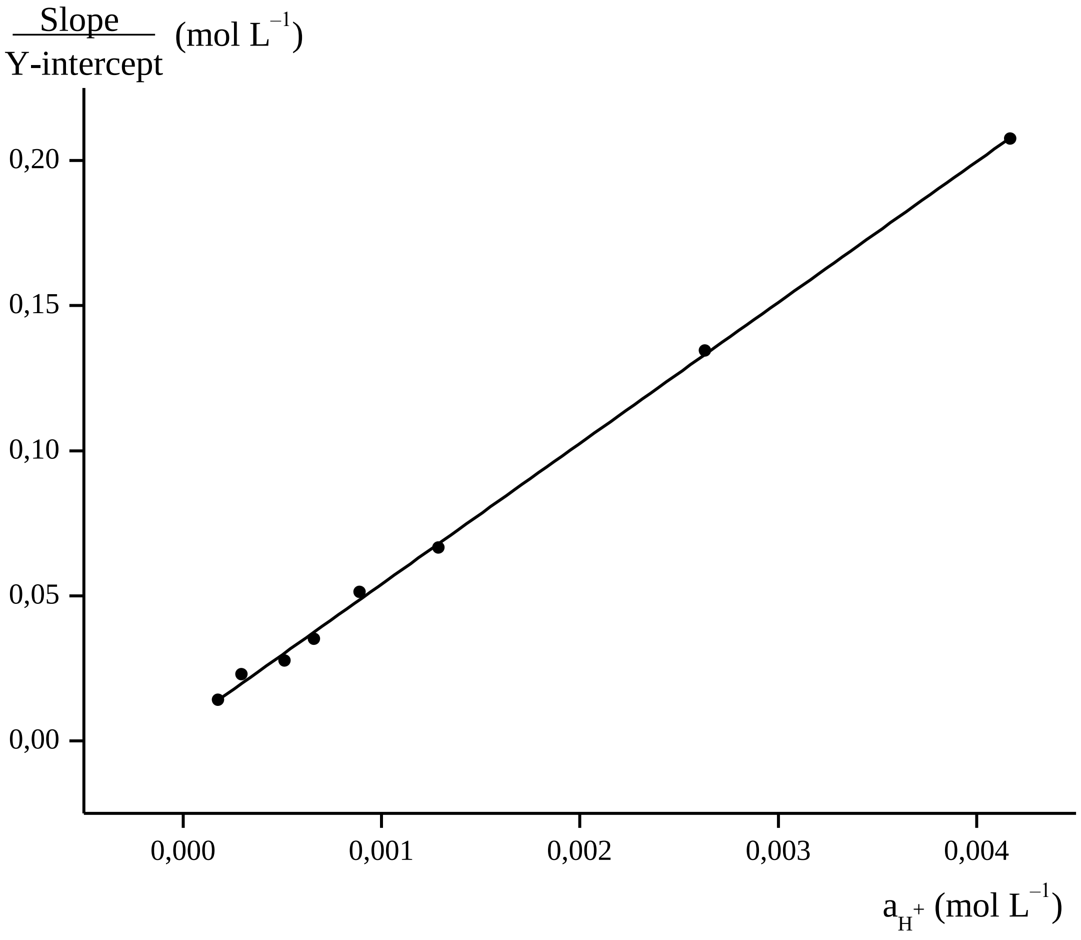

Figure S3. Determination of the k_{-1}/k_2 and k_{-2}/k_3 ratios using those of the slopes and Y-intercepts of the linear regressions shown in Fig. S1. The slope of a given linear regression is equal to $k_{-1}k_{-2}/k_2k_3$ and its Y-intercept is equal to k_{-1}/k_2. The value of $k_{-2}/\ k_3$ was calculated as the corresponding slope-to-Y-intercept ratio.

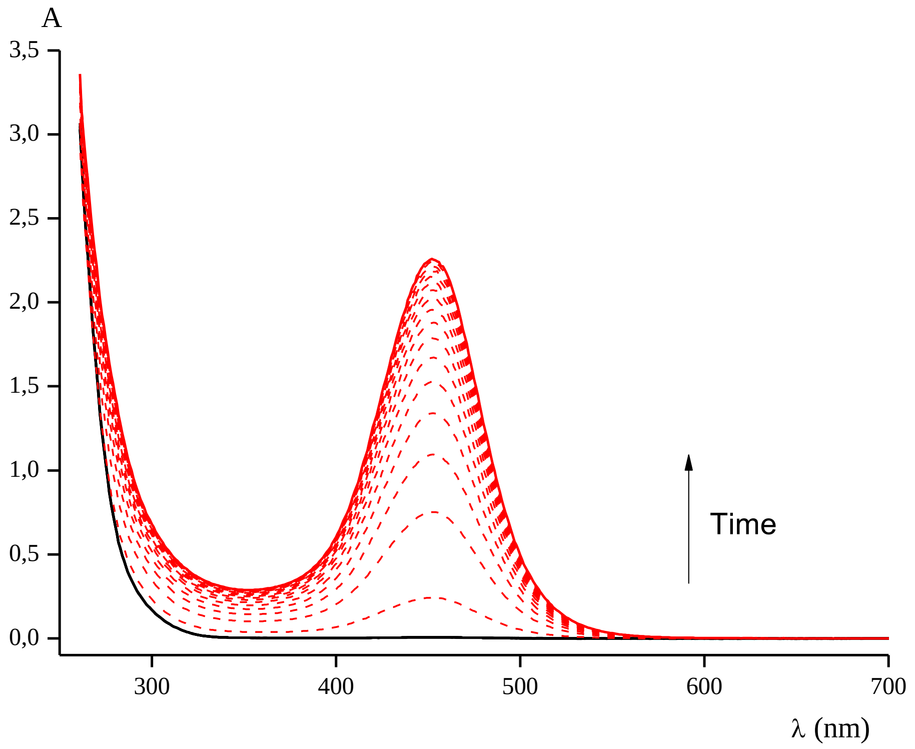

Figure S4. UV-vis spectrum of the initial mixture of all the reagents except of Fe^{2+} ions (shown in black) and those after their addition (shown in red, a time evolution caused by formation of the complex $FeOx_3(BCH_3)_2$ (**5**)).

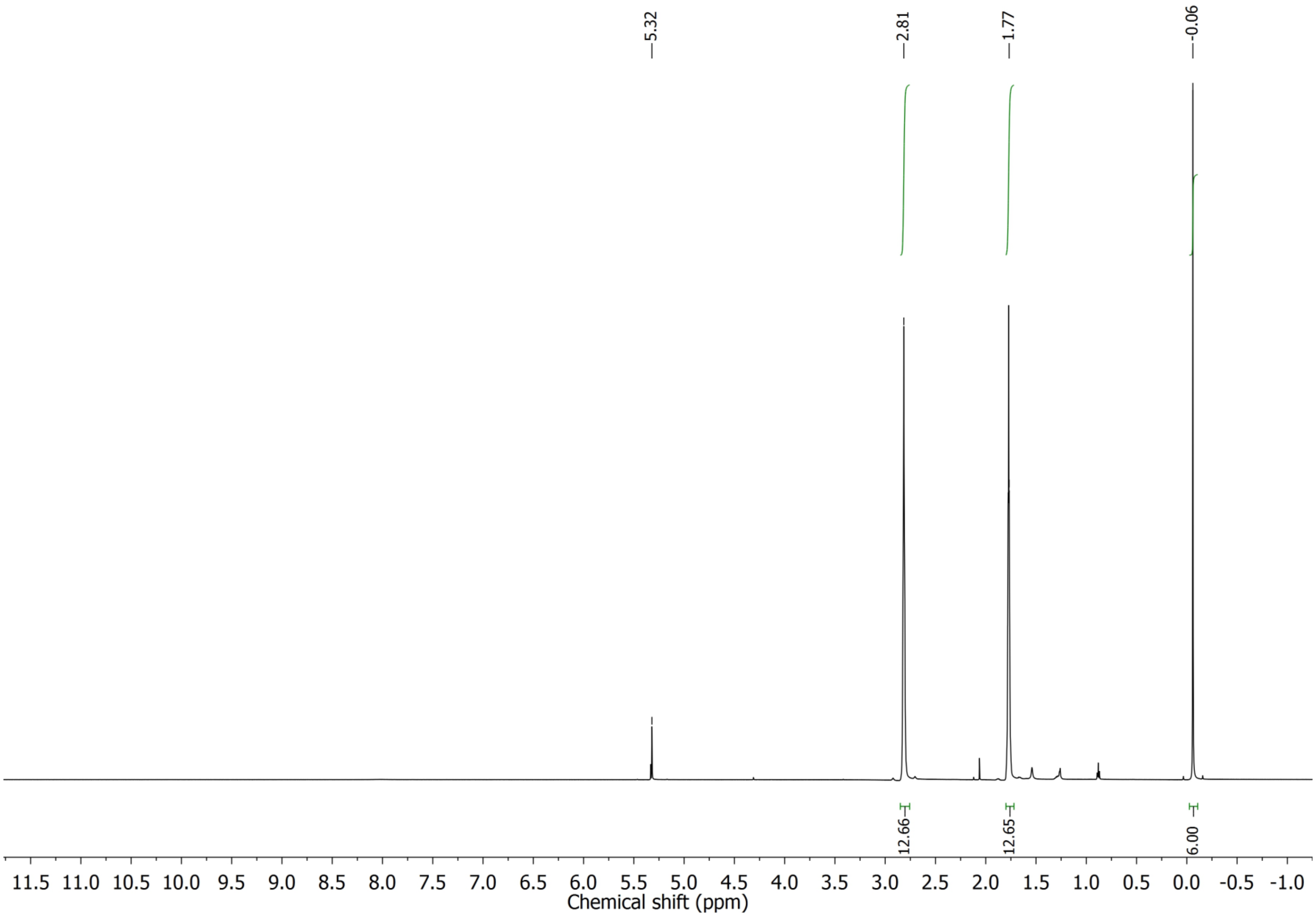

Figure S5. ^{1}H NMR spectrum of the complex $FeNx_3(BCH_3)_2$ (**4**) in CD_2Cl_2.

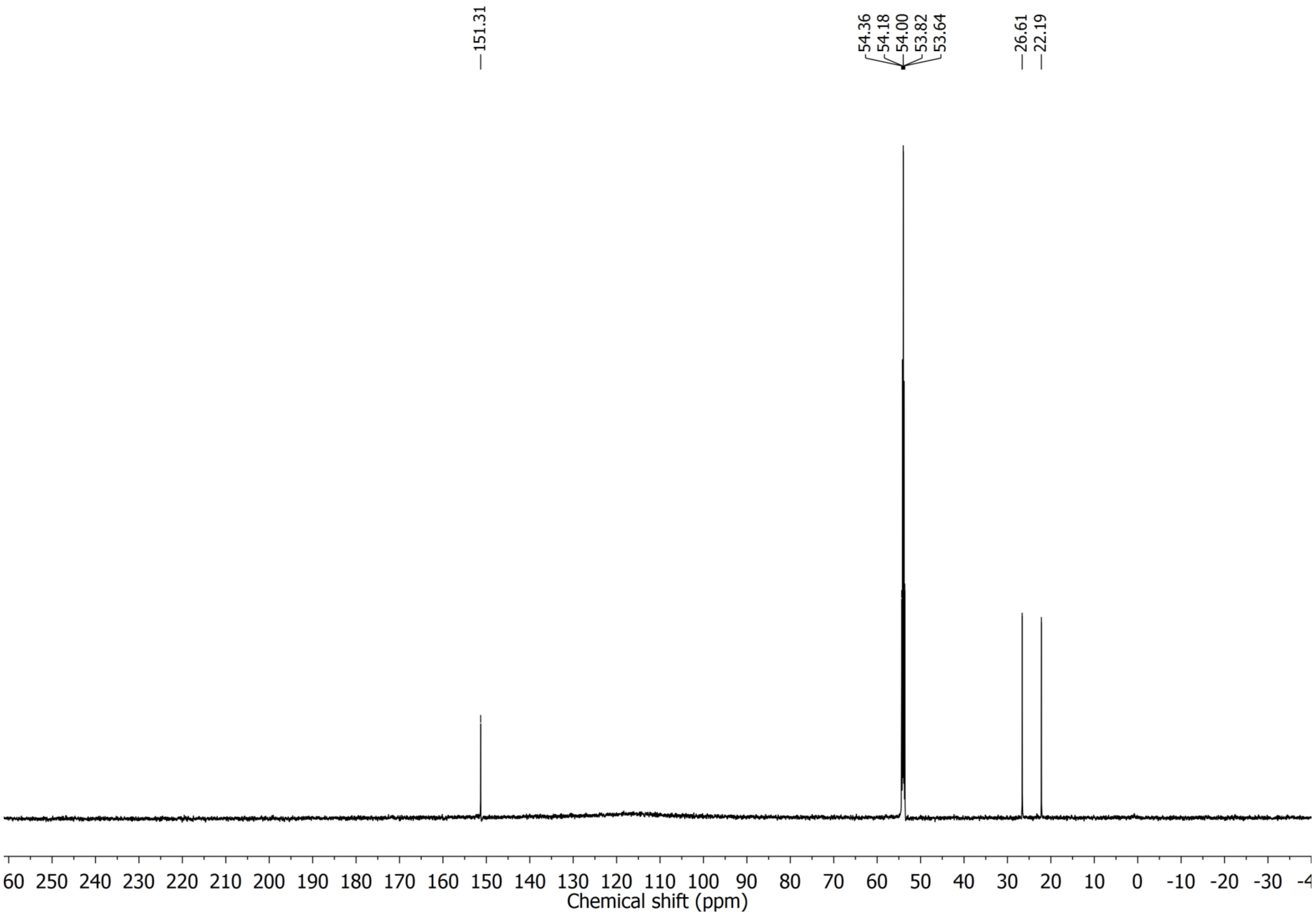

Figure S6. $^{13}C\{^{1}H\}$ NMR spectrum of the clathrochelate $FeNx_3(BCH_3)_2$ (**4**) in CD_2Cl_2.

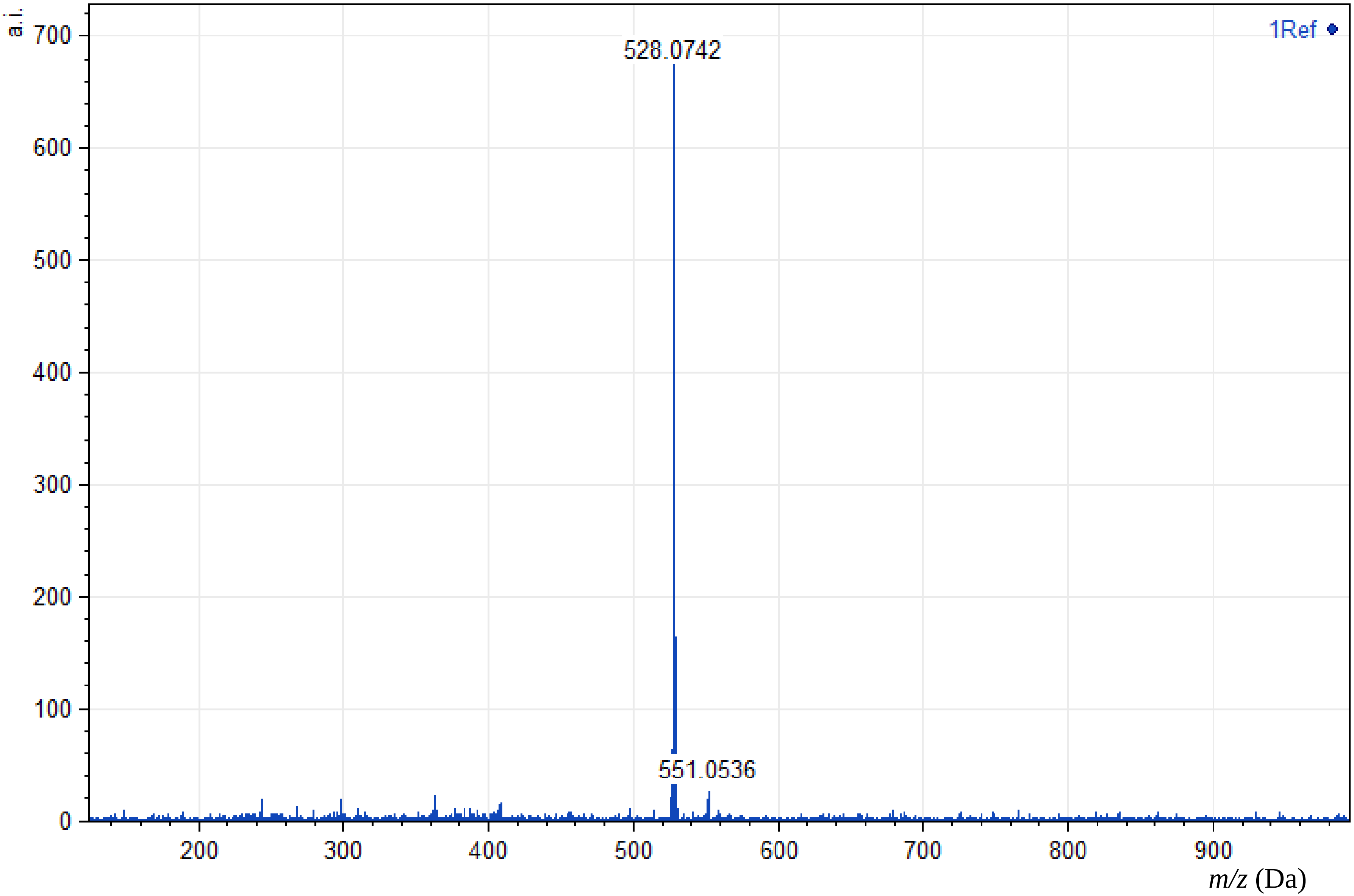

Figure S7. Positive range of MALDI-TOF spectrum of the complex $FeNx_3(BCH_3)_2$ (**4**).

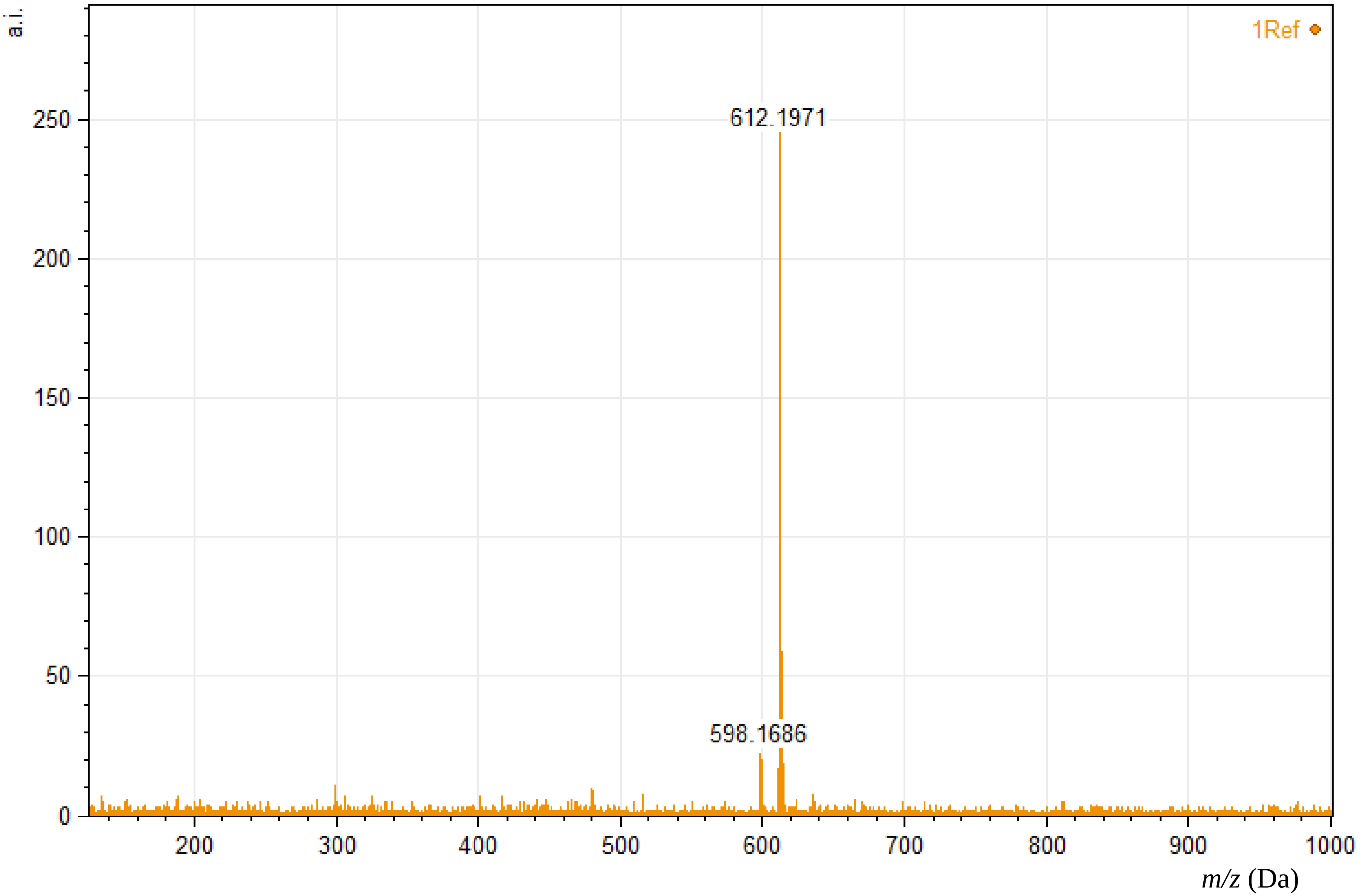

Figure S8. Positive range of MALDI-TOF spectrum of the complex $FeOx_3(BCH_3)_2$ (**5**).

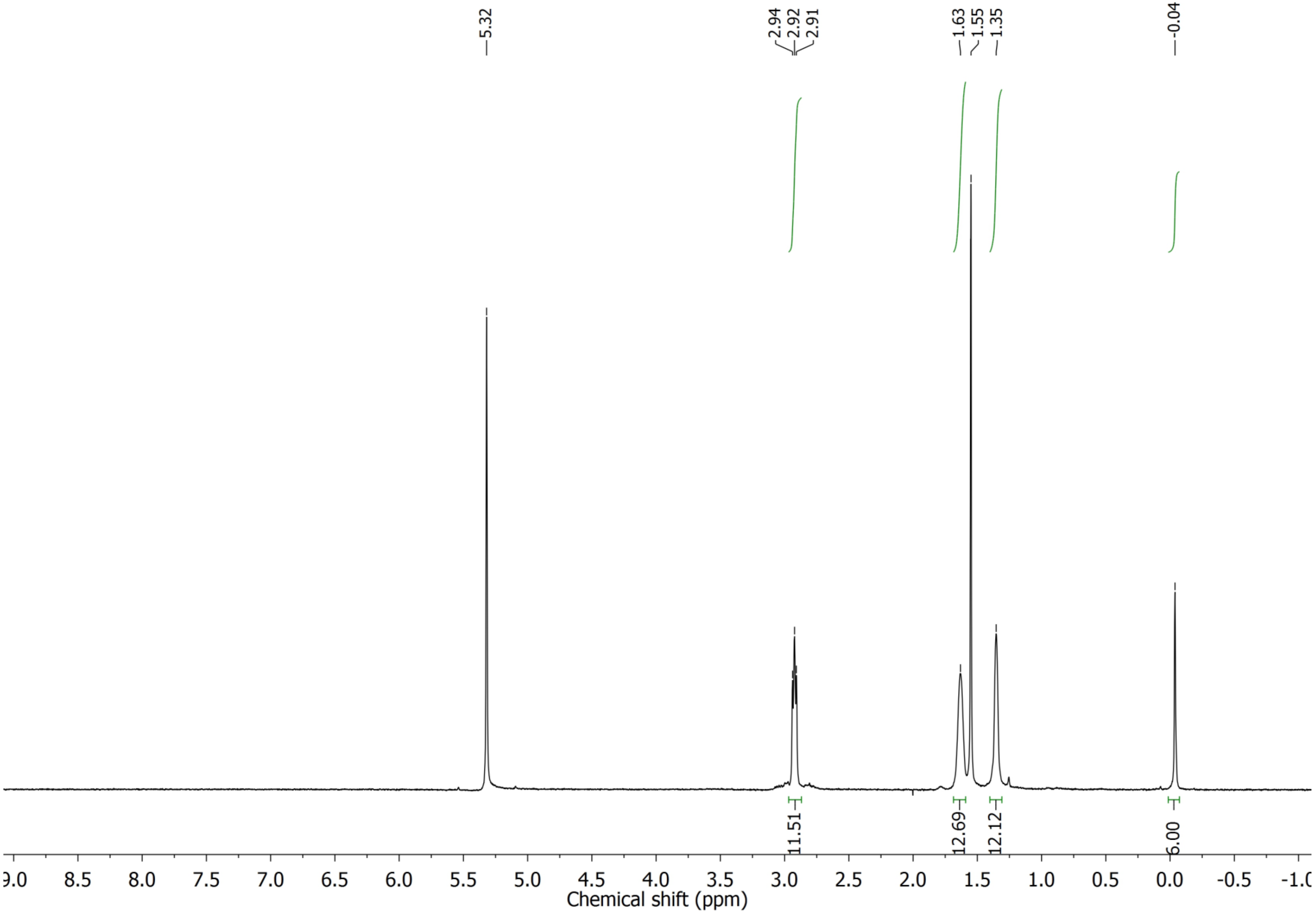

Figure S9. ^{1}H NMR spectrum of the complex $FeOx_3(BCH_3)_2$ (**5**) in CD_2Cl_2.

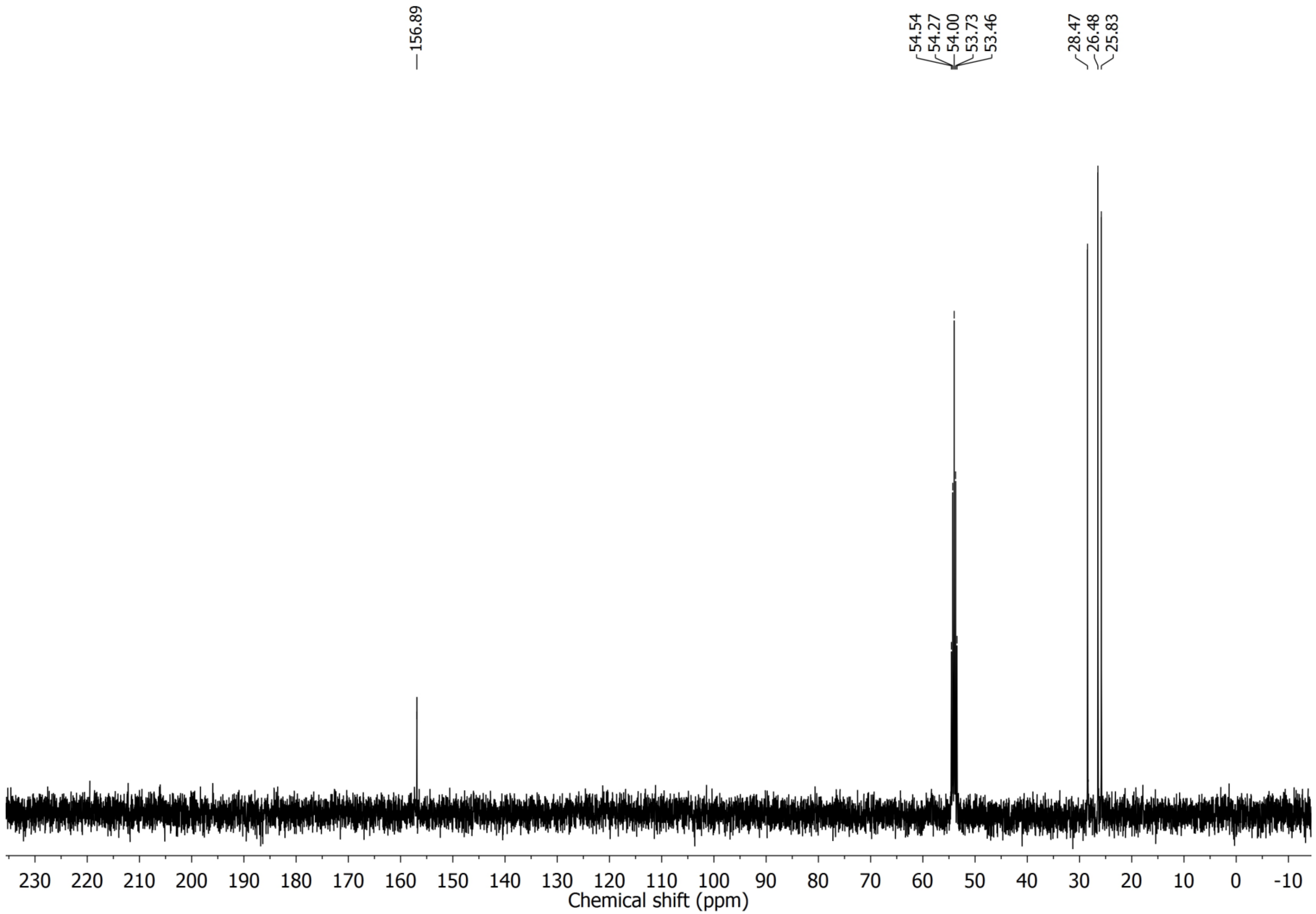

Figure S10. $^{13}C\{^{1}H\}$ NMR spectrum of the clathrochelate $FeOx_3(BCH_3)_2$ (**5**) in CD_2Cl_2.
